# Injury Severity Influences Long‐Term Cognitive Control in Pediatric “Mild” Traumatic Brain Injury

**DOI:** 10.1002/hbm.70580

**Published:** 2026-07-03

**Authors:** Upasana Nathaniel, Tracey V. Wick, Josef L. Ling, Vadim Zotev, Divyasree Sasi Kumar, Samuel D. Miller, Harm J. van der Horn, John P. Phillips, Andrew R. Mayer

**Affiliations:** ^1^ The Mind Research Network/Lovelace Biomedical Research Institute Albuquerque New Mexico USA; ^2^ University of Groningen, University Medical Center Groningen Groningen the Netherlands; ^3^ Department of Neurology University of New Mexico Albuquerque New Mexico USA; ^4^ Department of Psychology University of New Mexico Albuquerque New Mexico USA

**Keywords:** cognitive control, mild traumatic brain injury, pediatrics, task fMRI

## Abstract

Pediatric “mild” traumatic brain injury (pmTBI), or concussion, is increasingly associated with persisting and often undiagnosed cognitive difficulties, particularly, in adolescents. Emerging evidence suggests that these deficits are most pronounced during tasks requiring competing attentional demands (i.e., reactive control). However, it remains unclear how injury severity, indexed by loss of consciousness and/or posttraumatic amnesia (LOC/PTA) interacts with neurodevelopment to shape long‐term, modality‐specific alterations in cognitive control. In this longitudinal fMRI study, we examined reactive cognitive control using a multimodal attention task in a large cohort of pmTBI (*N* = 236) and age‐ and sex‐matched healthy controls (*N* = 212), across three timepoints (~1 week, ~4 months, and ~1 year) postinjury. Participants with LOC/PTA+ exhibited reduced task performance across both visual and auditory modalities, accompanied by persistent neural disruptions, indicating that greater injury severity is associated with more widespread, cross‐modal impairments in attentional control. In contrast, participants without LOC/PTA− demonstrated increased neural activation early after injury, potentially reflecting compensatory recruitment in the auditory domain to maintain performance. However, this was accompanied by greater clinical burden (i.e., sleep disturbance, depression) and was followed by evidence of dysregulated control by ~1 year postinjury, suggesting that short‐term neural compensation may carry longer‐term consequences. Notably, neural differences across groups were primarily observed in the auditory domain, with alterations in frontotemporal and cerebellar regions. This modality‐specific effect may reflect developmental shifts toward visual dominance during adolescence, which could increase processing demands for auditory information and render it more vulnerable to disruption following injury. Together these findings indicate that pmTBI is associated with persistent alterations in cognitive control processes that evolve over time as a function of injury severity.

## Graphical abstract

Greater injury severity (i.e., presence of loss of consciousness and/or posttraumatic amnesia; LOC/PTA+) is associated with an increased risk of persistent, cross‐modal deficits in attentional control. Milder cases (LOC/PTA−) exhibit early compensatory neural recruitment that supports performance but is associated with greater symptom burden and long‐term dysregulation of cognitive control.

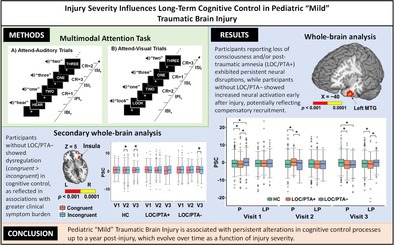

## 1 Introduction

Pediatric “mild”^1^ traumatic brain injury (pmTBI), also known as concussion (Silverberg et al. 2023), is highly prevalent in the United States, with sport‐ and recreation‐related injuries alone accounting for an estimated 1–2 million cases annually (Bryan et al. 2016; Taylor et al. 2017). Contrary to the assumption of rapid recovery, pmTBI has been associated with persistent alterations in brain development, including increased brain age and reduced hippocampal volumes up to a year postinjury (Mayer et al. 2015, 2023; Nathaniel et al. 2025). Because neural circuits supporting cognitive control continue to mature throughout childhood and adolescence, they remain, particularly, vulnerable to injury (Luna 2009). Even mTBI has been linked to ongoing and often undiagnosed cognitive problems (Hou et al. 2012; Jonsson et al. 2004; McInnes et al. 2017), with adolescents showing more persistent cognitive deficits than younger children or older adults (Baillargeon et al. 2012; Muscara et al. 2008; Tonks et al. 2011).

Recent evidence suggests that cognitive deficits after pmTBI are particularly evident on tasks requiring cognitive flexibility (Chadwick et al. 2021). Measures involving competing demands, such as dual‐task paradigms, have therefore been used to objectively track concussion recovery, for example, adolescents with concussion exhibit greater gait and balance impairments than uninjured peers when simultaneously performing complex cognitive tasks (Howell et al. 2014). These findings align with models of reactive cognitive control, which support the ability to resolve competing, stimulus‐driven demands and rely on sustained engagement of the cognitive control network (Braver 2012; Maki‐Marttunen et al. 2019). Reactive control therefore relies on the detection and resolution of high interference after its onset (Braver 2012), and is associated with ventrolateral prefrontal cortex (VLPFC), dorsal anterior cingulate cortex (dACC)/presupplementary motor area (preSMA), as well as with more transient dorsolateral prefrontal (DLPFC) recruitment (Braver et al. 2009; Irlbacher et al. 2014; Ryman et al. 2019). The VLPFC supports conflict resolution between competing stimuli (D'Esposito et al. 1999; Zandbelt et al. 2013), while the dACC/preSMA respond to increased error likelihood and sensory or response conflict (Carter and van Veen 2007; Shenhav et al. 2013).

Studies of mTBI report both hypo‐ and hyperactivation within the cognitive control network, with variability driven by injury stage and task demands, and evidence of a shift from early hypo‐ to a later hyperexcitable state during recovery (Mayer et al. 2014; Miller et al. 2014; Seeger et al. 2017). Consistent with this trajectory, recent work has demonstrated an inverse association between posttraumatic amnesia (PTA) duration and activation during reactive control within the first week postinjury in pmTBI (van der Horn et al. 2023). Notably, disruptions in reactive control were largely confined to the auditory rather than visual domain (van der Horn et al. 2023). Developmentally, children preferentially attend to auditory information until approximately 9 years of age, followed by a gradual shift toward visual dominance emerging in early adolescence (Barnhart et al. 2018; Sicard et al. 2021; Wille and Ebersbach 2016). However, most existing work has focused on typically developing children under 12, leaving adolescent development and longer‐term injury effects on reactive cognitive control relatively understudied. Together these findings highlight a critical gap in understanding how injury severity (i.e., loss of consciousness and/or posttraumatic amnesia [LOC/PTA]) and recovery stage interact with neurodevelopment to shape long‐term, modality‐specific alterations in cognitive control.

The current study uses a multimodal attention task to examine reactive control in a large cohort of pmTBI (*N* = 236) and an age‐ and sex‐matched group of healthy controls (HC; *N* = 212), across three timepoints: ~1 week (Visit [V]1), ~4 months (V2) and ~1 year (V3) postinjury. Consistent with disrupted network regulation early after injury (Mayer et al. 2014), we hypothesized that individuals with pmTBI would exhibit deficits in reactive control through 4 months postinjury, with normalization by 1 year, and that these changes would be driven by injury severity markers of LOC/PTA. Based on typical neurodevelopment and the natural dominance of the visual system, we expect individuals with pmTBI, particularly, those with LOC/PTA, to show persistent difficulty on incongruent auditory trials, reflected in slower reaction time and/or reduced accuracy together with increased activation, consistent with heightened susceptibility to distraction (van der Horn et al. 2023). Finally, we predict that in addition to injury‐related mechanisms, persistent deficits in clinical and cognitive assessments across visits would also be associated with altered brain functioning, which will be examined in secondary analyses.

## 2 Materials and Methods

### 2.1 Participants

Participants with pmTBI aged 8–18 years (*N* = 279, see Supporting Information and Figure S1) were consecutively recruited from local Emergency Departments and Urgent Care settings from July 2016 to December 2023. Inclusion criteria combined elements from the American Congress of Rehabilitation Medicine for “mild” traumatic brain injury and the Zurich Concussion in Sport Group criteria. Patients meeting the following criteria were enrolled: (1) a closed head injury with a Glasgow Coma Scale ≥ 13, (2) LOC (if present) limited to 30 min, (3) PTA (if present) limited to 24 h, (4) an alteration in mental status at the time of injury, or (5) presence of at least two new postconcussive symptoms. The first visit occurred within 11 days of injury (V1), second visit approximately 4 months (V2), and third visit 1 year (V3) postinjury. Age‐ and sex‐matched (*N* = 242) typically developing HC were recruited from the local community by word‐of‐mouth or fliers. The days between visits were consistent for both the HC and pmTBI groups to account for typical neurodevelopment and/or effects associated with repeat assessment. A subset of the data from the first two visits (77.4%) has already been published (van der Horn et al. 2023). General exclusion criteria for both participant groups were (1) presence of any neurological diagnosis, (2) previous TBI with LOC > 30 min, (3) developmental disorders (autism spectrum, or intellectual disability), (4) any psychiatric diagnosis other than adjustment disorder, (5) contraindications for MRI (including pregnancy and braces), (6) non‐English speaking, or (7) history of substance abuse/dependence. Participants with pmTBI were further excluded if general anesthesia was administered during routine trauma care, or if injury affected the dominant hand. For HC, additional exclusion criteria were a diagnosis of attention‐deficit/hyperactivity disorder or a learning disability. All participants underwent urine‐based drug screenings (see Supporting Information) and a positive drug screen also led to exclusion with the exception of recreational marijuana use. All procedures were approved by the University of New Mexico Health Sciences Human Research Review Committee (HRRC), and informed consent (i.e., ages 12–18) or assent (ages 8–11) was obtained from participants and their parents based on institutional guidelines.

### 2.2 Clinical Measures

A battery of Common Data Elements of clinical and neuropsychological tests (see Table 1 for primary and secondary measures; and Supporting Information and Table S1) was administered to patients and HC at all study visits. Primary clinical measures included age−/rater‐appropriate versions of the Post‐Concussion Symptom Inventory, Pediatric Quality of Life Inventory, and Conflict and Behavioral Questionnaire. Retrospective rating of symptoms 1 month before the initial visit were also collected (at V1 only). Patients with pmTBI were binarily classified with high PCS burden (i.e., symptomatic vs. asymptomatic) using a normative rather than simple change approach to reduce false positive rates (Mayer, Stephenson, et al. 2020). Secondary clinical measures included retrospective and current scales from the Patient‐Reported Outcomes Measurement Information System (sleep, anxiety and depression), self‐reported pain and headache ratings, Glasgow Outcome Scale Extended, and parent‐reported Strengths and Difficulties Questionnaire. Parental distress was further assessed using the Brief Symptom Inventory‐18. A semistructured interview was used to collect information on previous and current TBI history (Hergert et al. 2022). A comprehensive battery of neuropsychological tests measuring attention and processing speed were used as primary outcomes, whereas working memory, executive functioning, and long‐term memory recall were included as secondary outcomes. Additional neuropsychological tests assessing reading ability and effort were also collected.

**TABLE 1:** Central tendency data for clinical and neuropsychological measures.

Metric	V1 pmTBI (*N* = 236)	V1 HC (*N* = 212)	V2 pmTBI (*N* = 171)	V2 HC (*N* = 190)	V3 pmTBI (*N* = 144)	V3 HC (*N* = 147)
Symptom measures						
PCSI (% max)^b^, ^c^ (P)	15.9 (5.6–34.1)	2.9 (0–8.8)	4.8 (0–17)	3.2 (0.8–8.7)	4 (0.4–15.3)	2.7 (0–7.1)
PROMIS sleep^a^ (S)	19.1 ± 6.9	14.4 ± 4.8	18.3 ± 7.1	15.1 ± 4.9	18.5 ± 6.6	15.0 ± 5.0
PROMIS anxiety^a^, ^d^ (S)	3 (0–8)	1 (0–4)	2 (0–7.5)	1 (0–5)	2 (0–5)	0 (0–3)
PROMIS depression^a^ (S)	2 (0–10)	1 (0–4)	1 (0–7)	1 (0–4)	1 (0–6)	0 (0–4)
Pain scale^b^, ^c^ (S)	3 (1–5)	0 (0–1)	0 (0–3)	0 (0–1)	0 (0–2.5)	0 (0–1)
HIT‐6^b^ (S)	52 (44–60)	40 (36–46)	46 (40.5–55)	42 (38–48)	46 (40–53)	41 (38–48)
Behavioral and outcome measures						
CBQ^d^ (P)	1 (0–3)	1 (0–2)	1 (0–3)	1 (0–2)	1 (0–4)	0 (0–2)
PedsQL (P)	NA	NA	82.8 ± 13.4	87.1 ± 10.2	84.1 ± 3.1	88.8 ± 9.3
SDQ (S)	NA	NA	6 (4–10)	4 (2–7.5)	7 (3–9)	4 (2–6)
GOS‐E^b^ (S)	1 (1–4)	1 (1–1)	1 (1–1)	1 (1–1)	1 (1–1)	1 (1–1)
Cognitive measures						
TOMMe10^a^ (S)	10 (9–10)	10 (10–10)	10 (10–10)	10 (10–10)	10 (10–10)	10 (10–10)
WRAT4^a^ (S)	49.6 ± 10.3	56.4 ± 11.0	51.2 ± 11.3	58.2 ± 11.6	51.8 ± 10.5	57.7 ± 11.5
PS^b^ (P)	46.9 ± 7.7	50.4 ± 7.9	50.9 ± 8.2	53.0 ± 8.7	52.4 ± 8.3	55.4 ± 9.3
AT^b^ (P)	46.9 ± 8.6	51.4 ± 6.8	49.5 ± 7.7	52.0 ± 7.0	50.0 ± 7.0	52.6 ± 7.2
WM^a^ (S)	47.0 ± 7.8	50.5 ± 10.1	47.7 ± 9.0	51.6 ± 10.7	47.7 ± 8.6	51.8 ± 10.3
EF^a^ (S)	47.2 ± 7.2	50.6 ± 6.5	50.6 ± 6.8	53.4 ± 6.1	52.5 ± 6.2	54.7 ± 6.8
HVLT delay^a^, ^c^ (S)	7.6 ± 2.6	8.8 ± 2.0	7.6 ± 2.3	8.7 ± 2.3	8.3 ± 2.3	9.6 ± 1.9

*Note:* Outcomes are classified as primary (P) or secondary (S). Data are either formatted at mean ± standard deviation or median (interquartile range) based on distribution properties.

Abbreviations: AT = attention; CBQ = Conflict Behavior Questionnaire; EF = executive function; GOS‐E = Glasgow Outcome Scale Extended; HC = healthy control; HIT‐6 = Headache Impact Test; HVLT Delay = Delayed recall on Hopkins Verbal Learning Task (measure of long‐term memory); PCSI = Postconcussion Symptom Inventory (presented as percent of maximum score to account for age‐related scale differences); PedsQL = Pediatric Quality of Life Inventory; pmTBI = pediatric “mild” traumatic brain injury; PROMIS = Patient Reported Outcomes Measurement Information System; PS = processing speed; SDQ = Strengths and Difficulties Questionnaire; TOMMe10 = Test of Memory Malingering—10‐item short version; V1 = Visit 1 (~1 week postinjury); V2 = Visit 2 (~4 months postinjury); V3 = Visit 3 (~1 year postinjury); WM = working memory; WRAT4 = Wide Range Achievement Test 4.

^a^ Group main effect.

^b^ Group × Visit interaction.

^c^ Group × Age interaction.

^d^ Group × Visit × Age interaction.

### 2.3 fMRI Task Paradigm

**FIGURE 1:**
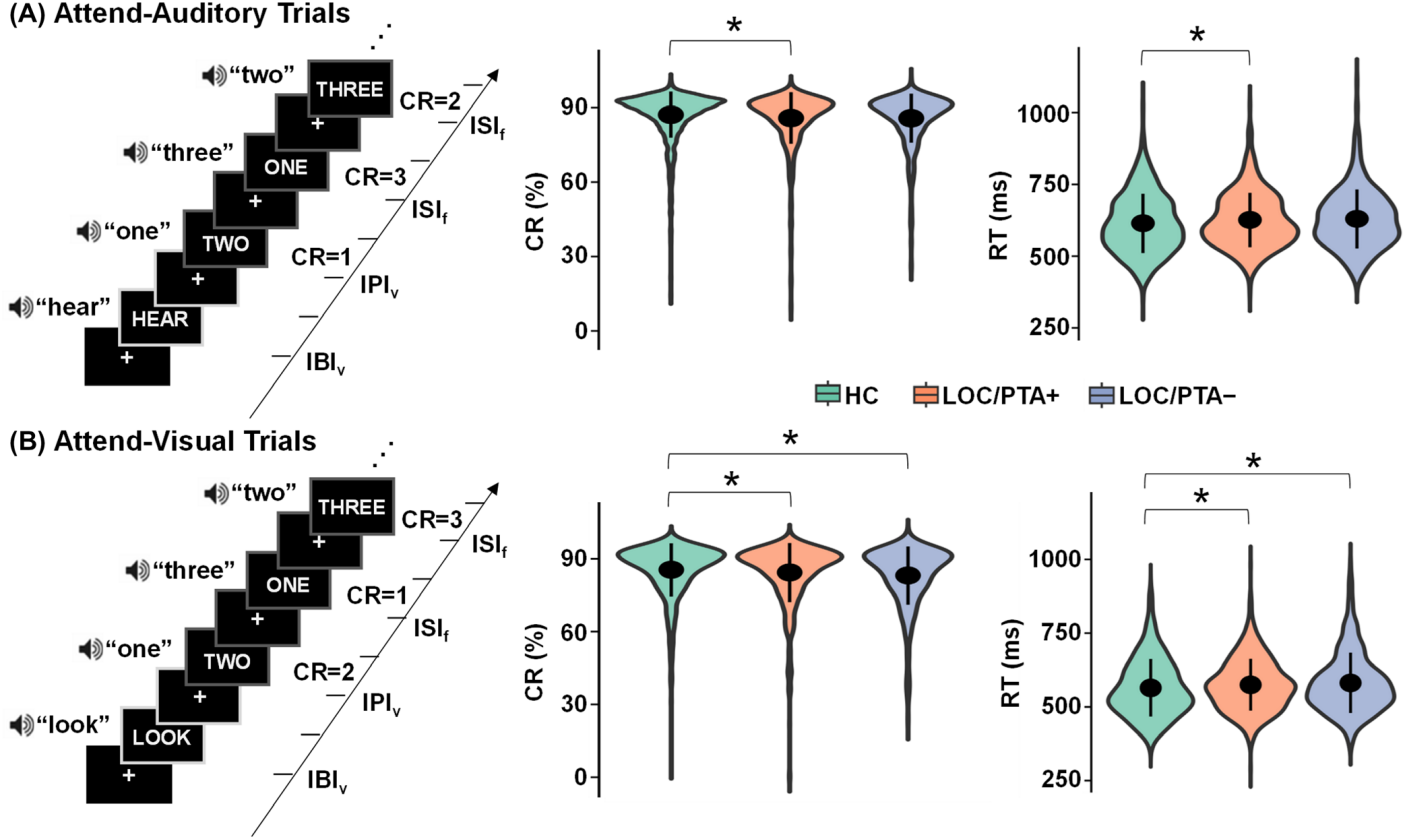
Diagrammatic representation of task and behavioral results. Participants attended to target stimuli in either the auditory (A) or visual (B) modality while ignoring congruent or incongruent stimuli in the opposite modality, as indicated by the correct response (CR). Each trial began with a multisensory cue (“HEAR” or “LOOK”) and was followed by a series of multisensory probes (numbers: one, two, or three). Separate hemodynamic response functions were derived for cues and probe phases through the inclusion of variable interphase intervals (IPI_v_) and interblock intervals (IBI_v_). The interstimulus interval between numeric targets was fixed (ISI_f_). Violin plots depict mean with standard deviation for accuracy (CR%) and reaction time (RT) separately for each modality. Participants were subdivided based on the presence or absence of loss of consciousness and/or posttraumatic amnesia (LOC/PTA+; LOC/PTA−) and healthy controls (HC). Asterisks denote significant group differences (*p* < 0.05, uncorrected).

The multimodal attention task has been described in previous studies (Mayer et al. 2017; Sicard et al. 2021). Participants were asked to fixate on a centrally presented crosshair between successive probes, respond to visual or auditory stimuli with a right‐handed button press using one of three consecutive buttons, and disregard stimuli in the other non‐attended modality (see Figure 1). The task was practiced outside the scanner prior to acquisition. Each block began with a 300 ms multisensory audiovisual cue indicating the modality to which participants should attend (“HEAR” = Attend‐Auditory; “LOOK” = Attend‐Visual). During the Attend‐Auditory and Attend‐Visual conditions, multisensory numeric probes (“ONE,” “TWO,” “THREE”; 300 ms duration) were presented as either congruent (i.e., matching across auditory and visual modalities) or incongruent (i.e., non‐matching across modalities). Probes were presented foveally and binaurally at a rate of 0.66 Hz within 7.8 s (6 trials per block). Visual probes were displayed as written words. The task included seven blocks for each of four conditions: Attend‐Visual congruent, Attend‐Visual incongruent, Attend‐Auditory congruent, Attend‐Auditory incongruent. The delay between cue onset and the first numeric target was jittered (2460–3380 ms) to enable separate modeling of the hemodynamic response function (HRF) for cue and probe (i.e., reflecting theoretical reactive control) conditions. Inter‐block intervals also varied (3700–5540 ms) to reduce temporal expectations and minimize nonlinear summing of HRFs across cues and probes (Glover 1999). Overall, the design yielded a non‐singular/invertible matrix with only moderate collinearity.

### 2.4 MRI Acquisition

Image acquisition was performed using a 3T Siemens TrioTim or a PRISMA fit scanner with a 32‐channel head coil (see Supporting Information for detailed scanning parameters). High‐resolution T1‐weighted (voxel size = 1.0 mm^3^), T2‐weighted (voxel size varied across the TrioTim [1.1 × 1.1 × 1.5 mm, 2D T2‐TSE] and PRISMA fit [1.0 mm^3^, 3D T2‐SPACE] platforms), susceptibility‐weighted (SWI) sequence (voxel size = 1.0 × 1.0 × 1.5 mm), and fluid attenuated inversion recovery (voxel size = 0.8 × 0.8 × 3 mm). Scanner versions remained consistent for each participant. For the acquisition of 2 runs of task, a single‐shot gradient‐echo‐echoplanar pulse sequence with 56 interleaved slices for whole‐brain coverage (3.02 × 3.02 × 3.00 mm^3^) was used, with multiband imaging (multiband factor = 8; TR = 460 ms) to allow for high temporal sampling of the HRF. All structural scans were reviewed by a blinded, board‐certified neuroradiologist for radiological common data elements (Mayer, Cohen, et al. 2020). A subset of pmTBI participants (*N* = 121) underwent computed tomography (CT) scans as part of routine care.

### 2.5 Imaging Processing

Task‐based fMRI data were despiked, slice timing corrected, and spatially registered in 2D and 3D to a single‐band reference image using tools from the AFNI software suite (Cox 1996). Susceptibility‐induced distortions were then estimated and corrected using the *topup* function in FSL, using two scans acquired with reversed‐phase encoding directions (Andersson et al. 2003; Smith et al. 2004). Images were subsequently normalized to Talairach standard space and spatially smoothed using a 6 mm FWHM kernel in AFNI.

Finite impulse response deconvolution was used to estimate a single HRF for the stimuli (20.7 s postonset) condition. First‐level models included the following nuisance regressors: motion parameters (three rotational and three translational) and their derivatives, error trials, and a second‐order polynomial per run to account for fMRI baseline effects. Beta‐weights were then summed and scaled to the average model intercept to derive percent signal change (PSC) for peak (8–17 s) and late peak (18–27 s) activity during the task.

### 2.6 Statistical Analyses

All clinical and demographic data were analyzed using the Statistical Packages for Social Sciences (SPSS; IBM Corp. Released 2011. IBM SPSS Statistics for Windows, Version 20.0. Armonk, NY: IBM Corp.). Generalized linear models (GLM; including Group effect only) or generalized estimating equations (GEE; including Group and Visit effects) with Gaussian, gamma or negative binomial distributions were used. All models included age‐at‐injury (in months) and retrospective ratings (if available) as covariates. Age‐at‐injury corresponds to chronological age for pmTBI (evaluated within 11 days postinjury) and to age at V1 for HC. Results were considered significant at *α ≤* 0.05 with Bonferroni corrections. Clinical outcomes were categorized into one of four patterns: complete recovery (Group × Visit interaction with no significant group effects at V3), partial recovery (Group × Visit interaction with reduced but statistically significant group differences at V3), no recovery (main effect of Group), or no deficits (absence of either main effect of Group or Group × Visit interaction).

#### 2.6.1 Behavioral Task Performance

Behavioral task data (i.e., reaction times and accuracy) were modeled using three‐way GEE with gamma distributions and unstructured covariance matrix [Group (LOC/PTA+ vs. LOC/PTA− vs. HC) × Visit (V1 vs. V2 vs. V3) × Congruency (Congruent vs. Incongruent)] and age‐at‐injury as a covariate in SPSS. Results were considered significant at the conventional statistical level of *p ≤* 0.05.

#### 2.6.2 Whole‐Brain Group fMRI Analyses

Imaging analyses were separated based on Attend‐Visual and Attend‐Auditory conditions, based on previously published data (Sicard et al. 2021). Only model terms that included Group were considered of interest in the current study. Whole‐brain linear mixed effects (LME) models were conducted using AFNI's 3dLME2 function (Chen et al. 2013) and R Core Team 2021. The source code was manually adjusted to specify an unstructured covariance matrix to correspond to clinical analyses. Results were corrected for family‐wise error (FWE) using a voxel‐wise statistical threshold of *p* < 0.001 (two‐tailed) and a minimum cluster volume of 658 μL, which was determined using Monte Carlo simulation (10,000 iterations) and spherical autocorrelation estimates (Cox et al. 2017).

The following four‐way LME model was run separately for Attend‐Visual and Attend‐Auditory modalities (Group [LOC/PTA+ vs. LOC/PTA− vs. HC] × Visit [V1 vs. V2 vs. V3] × Congruency [Congruent vs. Incongruent] × Phase [Peak vs. Late Peak]), with an unstructured covariance matrix. Mean framewise displacement and scanner were included as nuisance variables. Results were considered significant at FWE *p ≤* 0.05.

Secondary analyses examined associations between whole‐brain clusters and clinical and cognitive measures that showed no evidence of recovery, using the same LME models as the imaging analyses, with mean‐centered clinical ratings and cognitive scores entered as covariates in separate models.

## 3 Results

### 3.1 Demographics and Clinical Characteristics

Following protocol exclusions, the final sample at each visit (see Figure S1) was determined based on participants who completed the multimodal attention task, passed quality assurance checks (QC), were not an outlier based on motion (mean framewise displacement > 3 × interquartile range), task performance (i.e., accuracy ≤ 0.399 on two or more of the four behavioral conditions, based on a binomial distribution), reaction time > 3 × interquartile range, and based on voxel‐wise activations (three standard deviations above the group mean on five or more of the eight imaging conditions).

Final analyses therefore included 236 pmTBI (105 females; age 14.7 ± 2.7; 7.4 ± 2.2 days postinjury) and 212 HC (96 females; age 14.3 ± 2.8) at V1. A total of 171 pmTBI (73 females; 131.5 ± 14.5 days postinjury; 124.2 ± 14.3 days between V1 and V2) and 190 HC (83 females; 126.9 ± 16.7 days between V1 and V2) completed V2, and 144 pmTBI (65 females; 368.7 ± 30.7 days postinjury; 363.8 ± 28.9 days between V2 and V3) and 147 HC (64 females; 366.7 ± 29.5 days between V2 and V3) completed V3. Patients who did not complete all visits were included in all statistical models using available data.

The pmTBI and HC groups did not differ in terms of biological sex, age, handedness, postpubertal stage, or self‐reported Tanner stage of development (all *p*'s ≥ 0.05). The groups differed for self‐reported history of previous head injuries (*χ* ^2^ = 20.24, *p* = 1.5e−04; pmTBI = 19.9%; HC = 6.2%), parental self‐reported psychopathology (Wald‐*χ* ^2^(1) = 30.79, *p* = 2.9e−08; pmTBI > HC), socio‐economic status (Wald‐*χ* ^2^(1) = 22.35, *p* = 1.7e−04), premorbid reading ability (Wald*‐χ* ^2^(1) = 45.71, *p* = 1.4e−11; pmTBI < HC), and effort (Wald‐*χ* ^2^(1) = 21.02, *p* = 4.5e−06; pmTBI < HC). We, therefore, used both reading ability and effort as covariates in neuropsychological analyses. Seven pmTBI participants had a positive CT scan, and eight had traumatic lesions on standard structural MRI (Mayer, Cohen, et al. 2020). Demographics and injury characteristics data for the LOC/PTA+/− groups are presented in Table 2.

**TABLE 2:** Demographics and injury characteristic data.

	V1 LOC/PTA+ (*N* = 149)	V1 LOC/PTA− (*N* = 87)	V1 HC (*N* = 212)
Age	15.25 (13.08–17.08)	14.5 (12.67–16.67)	14.4 (12.4–16.5)
Sex (% female)	40.3%	51.7%	45.3%
Tanner stage of development	4 (3–4)	4 (3–4)	4 (2–4)
Parent BSI‐18^a^	3 (1–7)	3 (1–7)	1 (0–4)
Handedness (% right)	91.3%	88.5%	89.2%
pmTBI history^a^	18.8%	21.8%	6.2%
Socio‐economic status^a^			
Low (< $49,999)	45.6%	41.4%	24.1%
Middle ($50,000–$74,999)	13.4%	16.1%	18.9%
High (> $75,000)	34.2%	35.6%	49.5%
Refused/unknown	6.7%	6.9%	6.6%
Injury characteristics			
CT collected/positive	69.2%/6.1%	25.3%/4.5%	—
MRI collected/positive	100%/3.4%	100%/3.4%	—
Mechanism of injury			
Struck by object	11.4%	17.2%	—
Struck by person	24.8%	25.3%	—
Fall	18.1%	23.0%	—
MVC	33.6%	28.7%	—
Assault	6.7%	2.3%	—
Bicycle‐related	4.7%	3.4%	—
Other	0.7%	0%	—
Sport/recreation related	51.7%	59.8%	—

*Note:* Data are formatted as median (interquartile range) based on distribution properties.

Abbreviations: BSI = Brief Symptom Inventory‐18; HC = healthy control; MVC = motor vehicle crash; pmTBI = pediatric “mild” traumatic brain injury; V1 = Visit 1 (~1 week postinjury).

^a^ Group main effect.

### 3.2 Clinical Outcome and Neuropsychological Data

Self‐reported clinical outcomes and neuropsychological data are presented in Table 1. Primary clinical measures revealed a significant Group × Visit interaction for PCS severity. Follow‐up GLM analyses indicated partial recovery (see Table 3 for associated statistics), with PCS severity higher in pmTBI relative to HC at V1 (37.3% symptomatic), V2 (18.1% symptomatic), and V3 (19.4% symptomatic). In contrast, no significant effects were found for the primary measures of quality of life and self‐reported behavioral disturbances (*p*'s > Bonferroni corrected *α* = 0.0167), suggesting full recovery. Secondary clinical measures revealed significant Group×Visit interactions in the domains of headache, global TBI outcomes (GOS‐E), and pain, indicating complete recovery (all *p*'s < Bonferroni corrected *α* = 0.007). Finally, no evidence of recovery was observed for secondary measures of behavioral/emotional difficulties, sleep disturbance, anxiety, and depression across all visits (pmTBI > HC).

**TABLE 3:** Statistical results for clinical data.

Pattern	Metric	Measure	Group × Visit Model (2 × 3)				Sensitivity analyses V2 (*p*)		Sensitivity analyses V3 (*p*)	
			Effect (Wald‐*χ* ^2^; *p*)	V1 (*p*)	V2 (*p*)	V3 (*p*)	pmTBI	HC	pmTBI	HC
Complete	Clinical	Headache (S)	*G* × *V* (*χ* ^2^ *=* 53.41; *p =* 2.5e−12)	< 0e−36	0.079	0.097	0.274	0.567	0.599	0.651
		Global outcome (S)	*G* × *V* (*χ* ^2^ = 9.93; *p* = 0.007)	3.2e−12	0.002	0.035*	0.523	0.697	0.32	0.702
		Pain scale (S)	*G* × *V* (*χ* ^2^ = 26.75; *p* = 1.6e−06)	< 0e−36	0.003	0.013	0.254	0.658	0.486	0.711
	Cognitive	Attention (P)	*G* × *V* (*χ* ^2^ = 11.78; *p* = 0.003)	2.9e−04	0.206	0.202	0.677	0.011	0.388	0.013
		PS (P)	*G* × *V* (*χ* ^2^ = 8.79; *p* = 0.012)	0.007	0.325	0.121	0.423	0.516	0.825	0.221
Partial	Clinical	PCS (P)	*G* × *V* (*χ* ^2^ = 35.13; *p* = 2.4e−08)	< 0e−36	9.7e−06	8.4e−06	0.861	0.62	0.656	0.511
No recovery	Clinical	Sleep (S)	*G* (*χ* ^2^ = 32.11; *p* = 1.5e−08)				0.486	0.670	0.668	0.509
		Anxiety (S)	*G* (*χ* ^2^ = 20.88; *p* = 4.9e−06)				0.959	0.243	0.374	0.815
		Depression (S)	*G* (*χ* ^2^ = 11.17; *p* = 0.001)				0.884	0.592	0.679	0.845
	Cognitive	EF (S)	*G* (*χ* ^2^ = 12.80; *p* = 3.5e−04)				0.699	0.12	0.593	0.415
		WM (S)	*G* (*χ* ^2^ = 7.08; *p* = 0.008)				0.767	0.733	0.98	0.254
		Long‐term memory (S)	*G* (*χ* ^2^ = 30.87; *p* = 2.8e−08)				0.752	0.734	0.184	0.531
No deficits	Clinical	Behavioral disturbance (P)					0.103	0.793	0.018	0.452

*Note:* All significant main effects of Group exhibited opposite patterns for clinical (pmTBI > HC) relative to neuropsychological (pmTBI < HC) findings. Sensitivity analyses compared data from V1 for those participants who remained in the study versus attrition.

Abbreviations: EF: executive function; G: group; P: primary domain; PCS: postconcussive symptoms; PS: processing speed; S: secondary domain; V: visit; WM: working memory.

* Denotes *p*‐value not significant following Bonferroni correction, which were performed separately for primary and secondary clinical and cognitive domains.

For the primary neuropsychological domains, attention and processing speed indicated complete recovery. Secondary cognitive measures indicated continued deficits in executive function, working memory, and long‐term memory for pmTBI relative to HC across all other follow‐up visits.

Uncorrected sensitivity analyses comparing returning and non‐returning participants were negative for all clinical and cognitive measures (13 total tests in each group; see Table 3) with the exception of behavioral differences in pmTBI at V3 (returners < nonreturners), and attention in HC at both V2 and V3 (returners < nonreturners). See Supporting Information for effects associated with age and other factors.

### 3.3 Behavioral Task Performance

Figure 1 shows behavioral data (i.e., accuracy and reaction time) during performance on the multimodal attention task. For the Attend‐Auditory condition, a significant Group effect was observed for accuracy (Wald‐*χ* ^2^(2) = 8.20, *p* = 0.017), with a significant difference between patients with LOC/PTA+ (mean [*M*] = 0.85) and HC (*M* = 0.87), and no differences relative to the LOC/PTA− group (*M* = 0.86) with either of the two cohorts (see Figure 1a). A significant Group effect was also found for reaction time (Wald‐*χ* ^2^(2) = 6.33, *p* = 0.042), which indicated a similar significant difference between LOC/PTA+ (*M* = 628.5) and HC (609.5), and no differences relative to the LOC/PTA− (*M* = 624.2) group with either of the other groups.

For the Attend‐Visual condition, a significant Group effect was found for accuracy (Wald‐*χ* ^2^(2) = 11.52, *p* = 0.003), with a significant difference between HC (*M* = 0.88) relative to both LOC/PTA+ (*M* = 0.86) and LOC/PTA− (*M* = 0.85) groups, and no difference between the patient groups (see Figure 1b). Similarly, a significant Group effect was found for reaction time (Wald‐*χ* ^2^(2) = 8.62, *p* = 0.013), indicating significant differences between HC (*M* = 571.3) relative to both LOC/PTA+ (*M* = 589.9) and LOC/PTA− (*M* = 589.2), and no difference between the patient groups. All other significant main effects for both conditions are reported in the Supplemental Results.

### 3.4 Whole‐Brain Group fMRI Analyses

**FIGURE 2:**
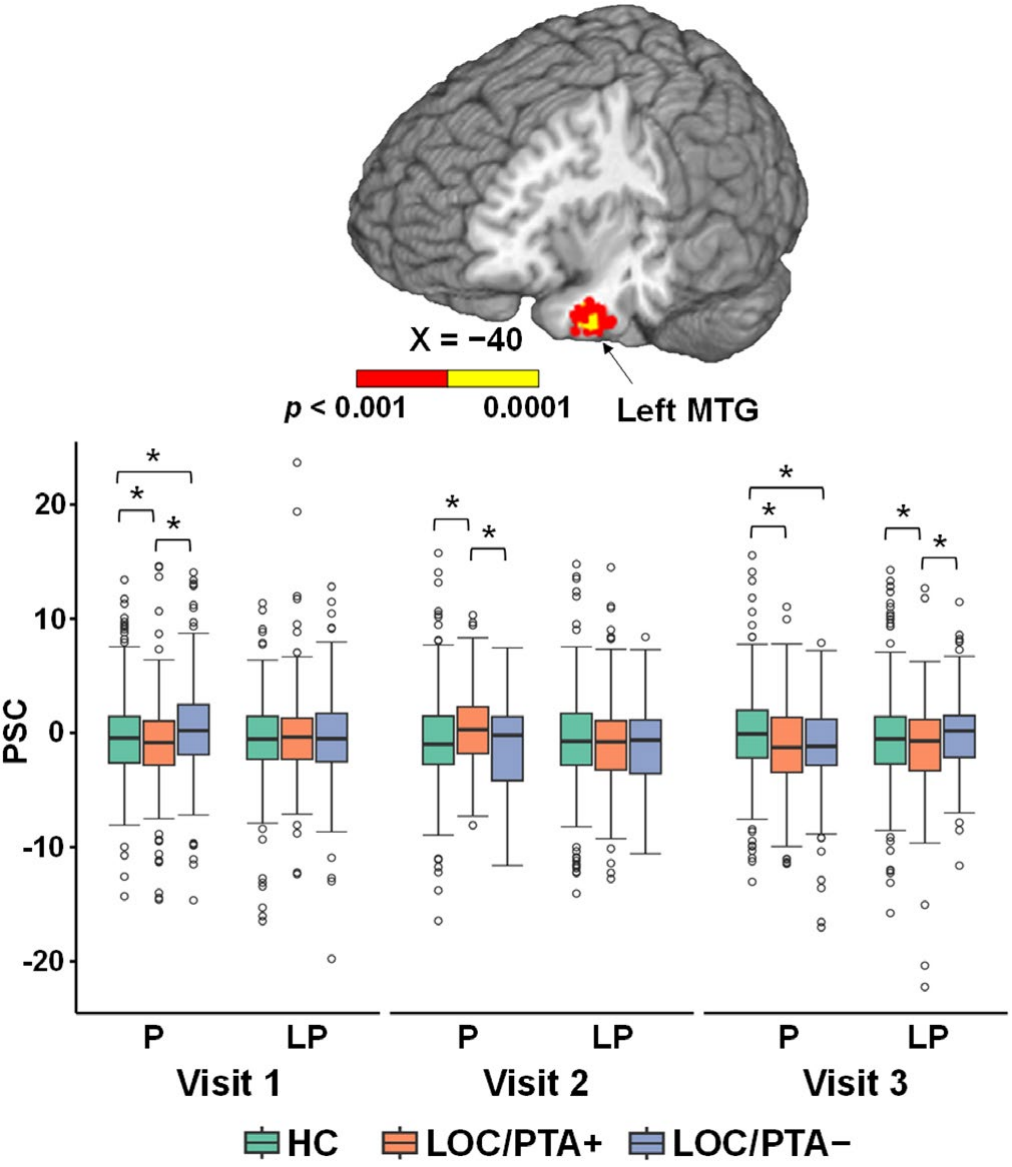
Results from whole‐brain voxel‐wise analysis for the Group × Visit × Phase interaction. Activation patterns in the left middle temporal gyrus (MTG) varied across participant subgroups based on the presence or absence of loss of consciousness and/or posttraumatic amnesia (LOC/PTA+; LOC/PTA−) relative to healthy controls (HC). In the peak (P) phase, the LOC/PTA− group indicated early hyperactivation relative to deactivation in LOC/PTA+ and HC at Visit 1 (~1 week postinjury), followed by greater activation in LOC/PTA+ at Visit 2 (~4 months postinjury), and subsequent deactivation in both patient groups relative to HC at Visit 3 (~1 year postinjury). The LOC/PTA+ group showed greater deactivation during the late peak (LP) phase at Visit 3, although this effect was sensitive to outlier removal. Asterisks denote significant (post hoc) comparisons. Location of the sagittal (*X*) slice is given according to the Talairach atlas.

There was no main effect of Group in either modality, but the following interactions with Group were observed in the Attend‐Auditory condition. A significant Group×Visit×Phase interaction was observed in the left middle temporal gyrus (BA21/38). Follow‐up LMEs revealed significant Group effects at all visits for the peak phase, and a Group effect for the late peak phase at V3 only (see Figure 2 and Table S2). During the peak phase at V1, activation differed across all groups with only the LOC/PTA− indicating positive activation (LOC/PTA− > HC > LOC/PTA+, all *p*'s < 0.05). At V2, the LOC/PTA+ group showed positive activation relative to deactivation in both HC (*p* = 0.001) and LOC/PTA− (*p* = 1.5e−04). At V3, HC exhibited positive activation relative to deactivation in both LOC/PTA+ (*p* = 3.8e−04) and LOC/PTA− (*p* = 0.002). For the late peak phase at V3, the LOC/PTA+ group showed greater deactivation than both LOC/PTA− (*p* = 0.016) and HC (*p* = 0.035); however, exclusion of the late peak V3 outlier eliminated this group difference.

**FIGURE 3:**
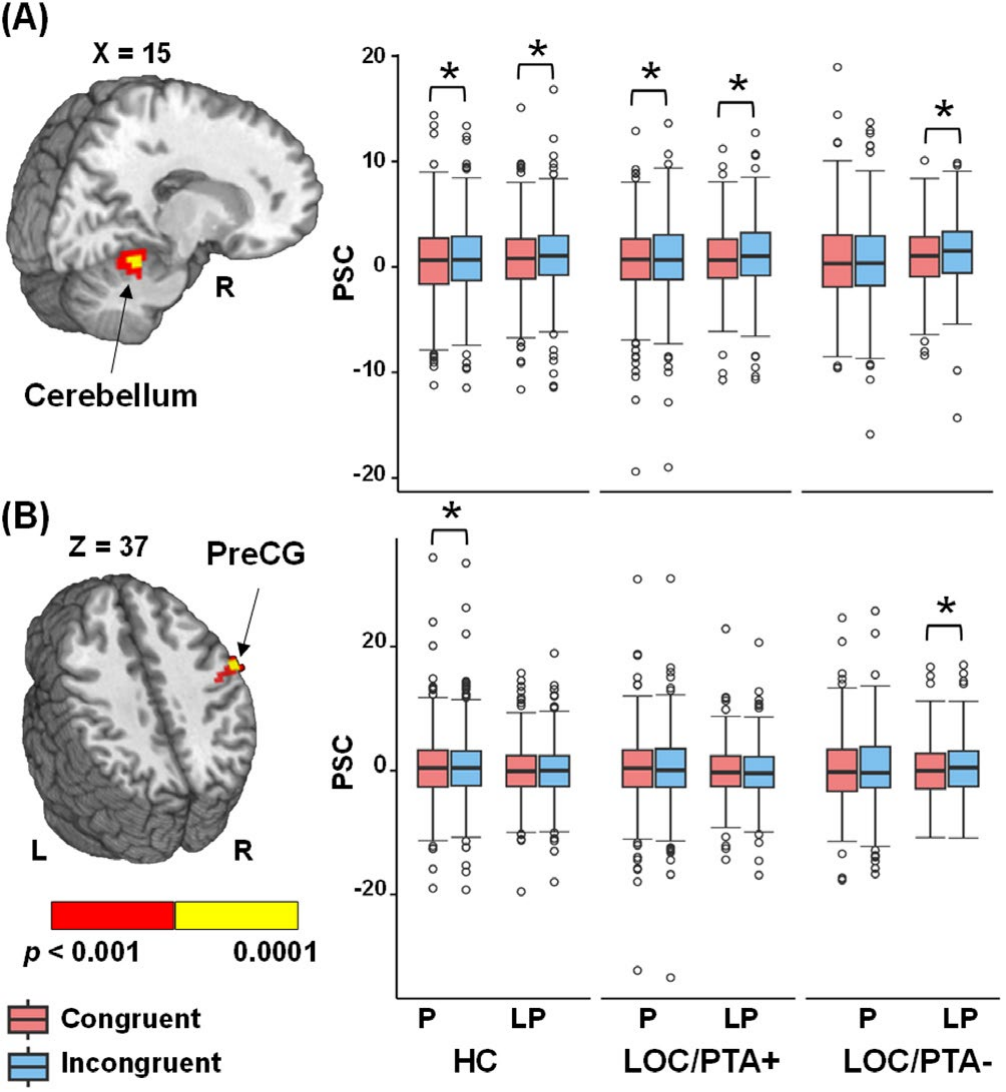
Secondary whole‐brain voxel‐wise analysis with clinical and cognitive covariates for the Group × Phase × Congruency interaction. Greater activation for incongruent > congruent trials was observed in the right cerebellum (A) and right precentral gyrus (PreCG; B). Healthy controls (HC) and participants reporting the presence or absence of loss of consciousness and/or posttraumatic amnesia (LOC/PTA+) showed this effect across the peak (P) and late peak (LP) phases, whereas the LOC/PTA− group showed it only in the late peak phase. Asterisks denote significant (post hoc) comparisons. Location of the sagittal (*X*) and axial (*Z*) slices is reported according to the Talairach atlas for the right (R) and left (L) hemispheres.

A significant Group × Phase × Congruency interaction was also observed in the Attend‐Auditory condition in the right cerebellum and right precentral gyrus (BA 6/4). Follow‐up analyses conducted separately by Group revealed a main effect of Phase in both clusters (see Figure 3 and Table S3). In the right cerebellum, this effect was significant for HC and LOC/PTA− (late peak > peak, both *p*'s < 0.038) but not for LOC/PTA+. In the right precentral gyrus, a Phase effect was observed only for HC (peak > late peak, *p* = 0.003), with no other significant effects. There were no other significant effects or interactions when split by Phase or Congruency.

In the Attend‐Auditory condition, two clusters showing a Group × Visit × Congruency interaction were found in the right insula and left middle frontal gyrus. However, follow‐up LMEs revealed no significant effects in either cluster. No significant interactions involving Group were observed in the Attend‐Visual condition. All other significant main effects for both conditions are reported in Supplemental Results.

### 3.5 Secondary Analyses: Relationship With Clinical and Cognitive Measures

Clinical and cognitive measures that demonstrated limited recovery (see Table 3) were entered in the above LMEs as mean‐centered covariates in separate clinical (i.e., sleep disturbance, anxiety and depression) and cognitive models (i.e., working memory, long‐term memory and executive functioning). For the Group × Visit × Phase interaction in left MTG, group differences across all visits in the peak phase remained significant after controlling for cognitive performance. In contrast, the group effect at V1 in the peak phase was not significant after controlling for clinical ratings, with a significant and negative association with anxiety (*β* = −0.088, *p* = 0.048).

For the Group × Phase × Congruency interaction in the right cerebellum, significant interactions with Congruency emerged after controlling for clinical and cognitive performance (see Figure 3a and Table S3). In the cognitive model, this effect was significant for HC and LOC/PTA+ across all phases (incongruent > congruent), with additional positive associations with working memory for HC in the peak phase (*β* = 0.040, *p* = 0.009) and executive functioning for LOC/PTA+ in both peak (*β* = 0.074, *p* = 0.008) and late peak (*β* = 0.059, *p* = 0.018) phases. A main effect of congruency was also observed for LOC/PTA− in the late peak phase, with no additional significant effects. Similar congruency effects were present in the clinical model, alongside an additional negative effect of sleep disturbance for LOC/PTA− in the late peak phase (*β* = −0.100, *p* = 0.008).

The Group × Phase × Congruency interaction in the right precentral gyrus indicated similar congruency effects (incongruent > congruent) observed for HC in the peak phase and for LOC/PTA− in the late peak phase, across both clinical and cognitive models (see Figure 3b and Table S3). A negative association with working memory was significant for LOC/PTA+ (*β* = −0.056, *p* = 0.045), while positive associations with executive functioning were significant for both LOC/PTA+ (*β* = 0.069, *p* = 0.030) and LOC/PTA− (*β* = 0.092, *p* = 0.046) in the late peak phase, with no other significant effects in the clinical model.

**FIGURE 4:**
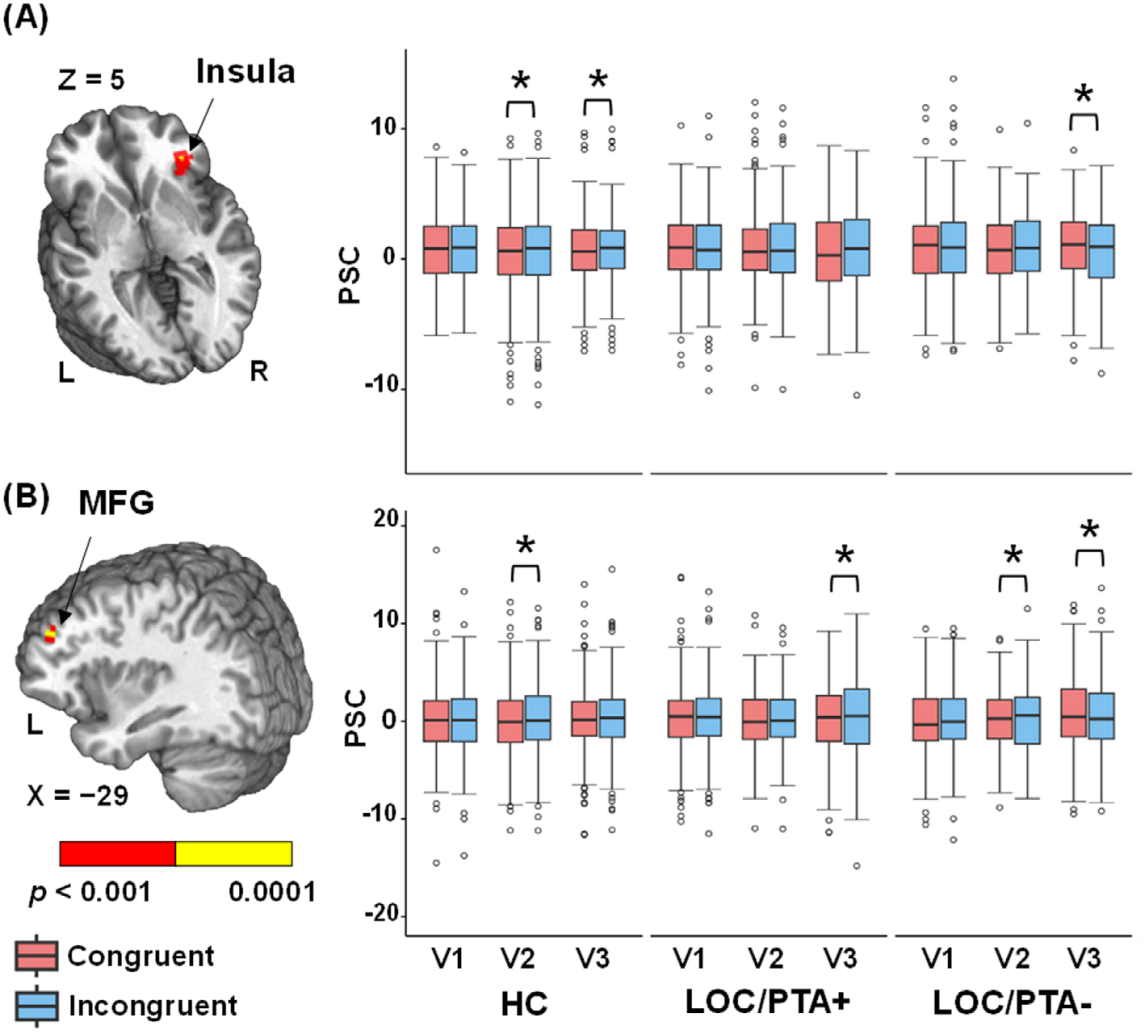
Secondary whole‐brain voxel‐wise analysis with clinical and cognitive covariates for the Group × Visit × Congruency interaction. Healthy controls (HC) and participant subgroups based on the presence or absence of loss of consciousness and/or posttraumatic amnesia (LOC/PTA+; LOC/PTA−) indicated greater activation for incongruent > congruent trials in the right insula (A) and left middle frontal gyrus (MFG; B) at ~4 months (V2) and ~1 year postinjury (V3) for HC and LOC/PTA+ groups. A reversal (congruent > incongruent) was observed only for the LOC/PTA− group at ~1 year postinjury (V3). Asterisks denote significant (post hoc) comparisons. Location of the sagittal (*X*) and axial (*Z*) slices is given according to the Talairach atlas for right (R) and left (L) hemispheres.

The Group × Visit × Congruency interaction in the right insula indicated interactions between Visit and Congruency when separated by Group (see Figure 4a and Table S4). A main effect of Congruency was observed for HC at Visits 2 and 3 (incongruent>congruent), and for LOC/PTA− at V3 (congruent > incongruent) across both cognitive and clinical models. A positive association with long‐term memory was significant for HC at V1 (*β* = 0.174; *p* = 0.023), while a negative association with working memory was significant for LOC/PTA− at V2 (*β* = −0.080; *p* = 0.018). Additional effects of a negative association with sleep disturbance (*β* = −1.130; *p* = 0.014) and a positive association with depression (*β* = 0.140; *p* = 0.031) were present only for LOC/PTA− at V1 in the clinical model.

Finally, for the Group × Visit × Congruency interaction in the left MFG (see Figure 4b and Table S4), a similar main effect of congruency (incongruent > congruent) was significant for HC at V2, LOC/PTA+ at V3, and LOC/PTA− at V2. The LOC/PTA− group also revealed a reversed congruency effect (congruent > incongruent) at V3 across both models. Additional effects were observed only in the LOC/PTA− group, showing a significant positive association with long‐term memory at V2 (*β* = 0.476; *p* = 0.003) and a negative association with sleep disturbance at V1 (*β* = −0.197; *p* = 3.4e−04).

## 4 Discussion

Using a multimodal attention task, the current longitudinal study suggests that reactive cognitive control following pmTBI is differentially affected by injury severity and ongoing clinical and cognitive symptoms. Across a large cohort of pmTBI and typically developing controls, the LOC/PTA+ group exhibited lower reaction times and reduced accuracy across visual and auditory domains, alongside persistent neural disruptions, suggesting more widespread cross‐modal deficits with greater injury severity. In contrast, the LOC/PTA− group showed early compensatory activation to maintain performance, but this was accompanied by greater clinical burden (i.e., sleep disturbance and depression) at V1 and evidence of dysregulated control by V3. Imaging findings were only observed in the auditory domain, with clusters in fronto‐temporal and cerebellar regions. These findings align with evidence of increased dominance of visual information, which may place greater demands in the processing of auditory stimuli in adolescents (Sicard et al. 2021; van der Horn et al. 2023). This vulnerability may be further exacerbated in children with pmTBI, consistent with evidence linking auditory symptoms, such as noise sensitivity, to broader cognitive impairments following mTBI (Faulkner et al. 2021; Hoover et al. 2017).

Despite stable task performance across visits, group‐specific alterations in brain activations were observed at various time points in the auditory domain. The LOC/PTA− group exhibited hyperactivation at V1 in the left MTG, implicated in auditory salience/processing (Braga et al. 2017), relative to deactivation in other groups. This group also showed increased activity in the right cerebellum (late peak > peak phase of the HRF), a region increasingly recognized for its role in higher‐order cognitive functions (Tenório et al. 2025). Hyperactivation following mTBI has been reported as a compensatory response to maintain task performance (Pardini et al. 2010; Urban et al. 2021), with recruitment of additional neural resources reflecting increased processing demands to achieve comparable behavioral performance (Bryer et al. 2013).

In contrast, the LOC/PTA+ group showed an unexpected pattern in the left MTG, with greater deactivation at 1 week followed by hyperactivation at 4 months relative to both groups and subsequent deactivation at 1 year relative to HC. Notably, LOC/PTA+ was the only group to exhibit reduced task performance in the auditory domain relative to both groups, suggesting this may reflect persistent neural disruption rather than the compensatory recruitment observed in the LOC/PTA− group. These findings parallel hypoactivation observed in left MTG in adolescents with prolonged exposure to subconcussive impacts (Talavage et al. 2014), suggesting potential similarities in participants exhibiting greater injury severity. Moreover, prior work has shown reduced hippocampal volume and increased cortical thickness at 4 months postinjury in patients with LOC/PTA+ (Nathaniel et al. 2025), raising the possibility that the transient hyperactivation observed at this timepoint may index ongoing structural alterations (Dall'Acqua et al. 2017; Hylin et al. 2017; Johnson et al. 2013) that disrupt efficient neural functioning.

It should also be noted that group differences in left MTG at V1 (i.e., deactivation in LOC/PTA+ and hyperactivation in LOC/PTA− relative to HC) were no longer significant after controlling for clinical symptoms, suggesting that variability in this region was at least partly related to anxiety, with higher anxiety associated with reduced activation. This interpretation is notable given the left MTG's role within the default mode network (DMN), which supports internally directed processes, including emotional processing (Buckner et al. 2008; Raichle 2015). Thus, the observed association between anxiety and reduced activation is consistent with evidence of altered DMN functioning in anxiety disorders (Sylvester et al. 2012; Zhao et al. 2023), and suggests that the left MTG may be, particularly, sensitive to both injury severity and symptom burden early after injury.

Although group and congruency interactions were not significant in the initial follow‐up analyses within the identified clusters, they were evident when controlling for ongoing clinical and cognitive disturbances. Across groups, the expected pattern of greater activation for incongruent > congruent trials was observed in the right cerebellum and precentral gyrus. In contrast, the LOC/PTA− group showed greater activation for congruent > incongruent trials in the right insula and left MFG at ~1 year postinjury relative to both groups, and was the only group to also exhibit associations with clinical measures in these two regions. Specifically, greater sleep disturbance was linked to reduced activation at 1 week in right insula and left MFG, while depressive symptoms were positively associated with insula activity, also at 1 week. This pattern suggests that early compensatory recruitment observed may carry downstream costs. The insula is implicated in salience detection and emotion processing (Craig 2009; Seeley et al. 2007), and alterations in insular activity postinjury may contribute to inefficient regulation of control over time. Despite preserved behavioral performance across congruency, activation engaged under higher task demands (incongruent) may not appropriately scale down when demands decrease (congruent), consistent with altered load‐dependent modulation and inefficient allocation of cognitive control resources in mTBI (Hammeke et al. 2013; McAllister et al. 2001). These findings are also in line with prior work demonstrating relatively intact ACC‐mediated conflict‐monitoring processes in adults with mTBI relative to controls (Larson et al. 2011), alongside greater vulnerability in DLPFC and related frontal systems supporting attentional control (Larson et al. 2012; Moore et al. 2014).

Relatedly, both patient groups also showed a negative association with working memory in the right insula (LOC/PTA−) and right precentral gyrus (LOC/PTA+), in contrast to a positive association observed in HC. This pattern suggests inefficient neural recruitment, whereby greater activation is required in individuals with poorer performance. These findings align with evidence of persistent working memory deficits up to 7 years after a mTBI (Moore et al. 2014), and with reports linking poorer working memory to altered activation in the DLPFC in adolescents with higher incidence of head injury (Talavage et al. 2014). In contrast, both patient groups demonstrated positive associations between executive functioning and activation in the right cerebellum and right precentral gyrus, suggesting that increased recruitment of these regions may support task performance following injury. Converging evidence indicates that the cerebellum plays an active role in modulating executive functioning through its connections with prefrontal and paralimbic regions, supporting the efficiency of higher‐order cognitive processes (Tenório et al. 2025). This pattern was, particularly, evident in the LOC/PTA+ group, which indicated positive associations with cerebellar activation across both peak and late peak phases.

A key strength of the study is the use of a multimodal attention task in a large cohort of adolescents, combined with a longitudinal design and identical (i.e., same battery and interval testing schedule) clinical and cognitive assessments across visits. This enabled a comprehensive characterization of cognitive control and recovery trajectories following pmTBI and allowed for the assessment of cross‐modal deficits that are particularly relevant during this developmental period. Second, the relatively large cohort, combined with age‐ and sex‐matched typically developing controls, enhances the generalizability and interpretability of the findings. However, although overall participant retention was high at 1 year postinjury (~77% across groups), it remained slightly below the optimal threshold (80%), which may limit sensitivity to longer‐term effects. Other limitation includes reliance on the BOLD signal. As with all fMRI studies, the BOLD signal is an indirect measure of neural activity (Hall et al. 2016) and susceptible to motion and other artifacts (Havsteen et al. 2017). While mean framewise displacement was included as a covariate in all imaging analyses, these factors may reduce its reliability and limit the ability to draw direct conclusions about underlying neural mechanisms. Future work integrating multimodal imaging and other objective measures could help to further clarify these relationships.

## 5 Conclusion

Current findings demonstrate that pmTBI is associated with persistent neural alterations up to a year postinjury, with recovery trajectories varying as a function of injury severity. While greater injury severity is associated with a higher risk of persisting cross‐modal deficits, even milder injury presentations may involve early compensatory processes that predict later dysregulation, as reflected in associations with greater clinical symptom burden. Notably, behavioral performance did not vary across visits despite persisting alterations in brain activation, highlighting the sensitivity of neuroimaging markers in detecting subtle but meaningful disruptions that may impact daily functioning.

## Funding

This research was supported by grants from the National Institutes of Health (https://www.nih.gov; grant numbers NIH 01 R01 NS098494–01A1, R01 NS098494–03S1A1, 2R01 NS098494‐06A1, and P30 GM122734) to Andrew R. Mayer. The NIH had no role in study review, data collection and analysis, decision to publish, or preparation of the manuscript. The content is solely the responsibility of the authors and does not necessarily represent the official views of the National Institutes of Health.

## Supporting information


**Figure S1:** Participant recruitment and retention.
**Figure S2:** Congruency effects in whole‐brain voxel‐wise analysis.
**Table S1:** Primary and secondary clinical and cognitive measures.
**Table S2:** Significant regions of activation for the Group × Visit × Phase interaction during the Attend‐Auditory condition of the multimodal attention task.
**Table S3:** Significant regions of activation for the Group × Phase × Congruency interaction during the Attend‐Auditory condition of the multimodal attention task.
**Table S4:** Significant regions of activation for the Group × Visit × Congruency interaction during the Attend‐Auditory condition of the multimodal attention task.

## Data Availability

The data that support the findings of this study will be openly available in FITBIR at fitbir.nih.gov upon the conclusion of the study, reference number FITBIR‐STUDY0000339.

## Endnotes

^1^
“Mild” TBI was classified according to published criteria (see Section 2) to maintain consistency with existing literature, while acknowledging that the term “mild” may not fully capture the underlying severity of injury (Silverberg et al. 2023).

## References

[hbm70580-bib-0001] Andersson, J. L. , S. Skare , and J. Ashburner . 2003. “How to Correct Susceptibility Distortions in Spin‐Echo Echo‐Planar Images: Application to Diffusion Tensor Imaging.” NeuroImage 20, no. 2: 870–888. 10.1016/S1053-8119(03)00336-7. 14568458

[hbm70580-bib-0002] Baillargeon, A. , M. Lassonde , S. Leclerc , and D. Ellemberg . 2012. “Neuropsychological and Neurophysiological Assessment of Sport Concussion in Children, Adolescents and Adults.” Brain Injury 26, no. 3: 211–220. 10.3109/02699052.2012.654590. 22372409

[hbm70580-bib-0003] Barnhart, W. R. , S. Rivera , and C. W. Robinson . 2018. “Different Patterns of Modality Dominance Across Development.” Acta Psychologica 182: 154–165. 10.1016/j.actpsy.2017.11.017. 29179020

[hbm70580-bib-0004] Braga, R. M. , P. J. Hellyer , R. J. Wise , and R. Leech . 2017. “Auditory and Visual Connectivity Gradients in Frontoparietal Cortex.” Human Brain Mapping 38, no. 1: 255–270. 10.1002/hbm.23358. 27571304 PMC5215394

[hbm70580-bib-0005] Braver, T. S. 2012. “The Variable Nature of Cognitive Control: A Dual Mechanisms Framework.” Trends in Cognitive Sciences 16, no. 2: 106–113. 10.1016/j.tics.2011.12.010. 22245618 PMC3289517

[hbm70580-bib-0006] Braver, T. S. , J. L. Paxton , H. S. Locke , and D. M. Barch . 2009. “Flexible Neural Mechanisms of Cognitive Control Within Human Prefrontal Cortex.” Proceedings of the National Academy of Sciences of the United States of America 106, no. 18: 7351–7356. 19380750 10.1073/pnas.0808187106 PMC2678630

[hbm70580-bib-0007] Bryan, M. A. , A. Rowhani‐Rahbar , R. D. Comstock , F. Rivara , and Seattle Sports Concussion Research Collaborative . 2016. “Sports‐and Recreation‐Related Concussions in US Youth.” Pediatrics 138, no. 1: e20154635. 27325635 10.1542/peds.2015-4635

[hbm70580-bib-0008] Bryer, E. J. , J. D. Medaglia , S. Rostami , and F. G. Hillary . 2013. “Neural Recruitment After Mild Traumatic Brain Injury Is Task Dependent: A Meta‐Analysis.” Journal of the International Neuropsychological Society 19, no. 7: 751–762. 10.1017/S1355617713000490. 23656706

[hbm70580-bib-0009] Buckner, R. L. , J. Andrews‐Hanna , and D. Schacter . 2008. “The Brain's Default Network: Anatomy, Function, and Relevance to Disease.” Annals of the New York Academy of Sciences 1124: 1–38. 18400922 10.1196/annals.1440.011

[hbm70580-bib-0010] Carter, C. S. , and V. van Veen . 2007. “Anterior Cingulate Cortex and Conflict Detection: An Update of Theory and Data.” Cognitive, Affective, & Behavioral Neuroscience 7, no. 4: 367–379. 10.3758/cabn.7.4.367 18189010

[hbm70580-bib-0011] Chadwick, L. , E. Roth , N. M. Minich , et al. 2021. “Cognitive Outcomes in Children With Mild Traumatic Brain Injury: An Examination Using the National Institutes of Health Toolbox Cognition Battery.” Journal of Neurotrauma 38, no. 18: 2590–2599. 10.1089/neu.2020.7513. 33906429 PMC8403208

[hbm70580-bib-0012] Chen, G. , Z. S. Saad , J. C. Britton , D. S. Pine , and R. W. Cox . 2013. “Linear Mixed‐Effects Modeling Approach to FMRI Group Analysis.” NeuroImage 73: 176–190. 10.1016/j.neuroimage.2013.01.047. 23376789 PMC3638840

[hbm70580-bib-0013] Cox, R. W. 1996. “AFNI: Software for Analysis and Visualization of Functional Magnetic Resonance Neuroimages.” Computers and Biomedical Research 29: 162–173. 8812068 10.1006/cbmr.1996.0014

[hbm70580-bib-0014] Cox, R. W. , G. Chen , D. R. Glen , R. C. Reynolds , and P. A. Taylor . 2017. “FMRI Clustering in AFNI: False‐Positive Rates Redux.” Brain Connectivity 7, no. 3: 152–171. 10.1089/brain.2016.0475. 28398812 PMC5399747

[hbm70580-bib-0015] Craig, A. D. 2009. “How Do You Feel—Now? The Anterior Insula and Human Awareness.” Nature Reviews. Neuroscience 10, no. 1: 59–70. 19096369 10.1038/nrn2555

[hbm70580-bib-0016] Dall'Acqua, P. , S. Johannes , L. Mica , et al. 2017. “Prefrontal Cortical Thickening After Mild Traumatic Brain Injury: A One‐Year Magnetic Resonance Imaging Study.” Journal of Neurotrauma 34, no. 23: 3270–3279. 10.1089/neu.2017.5124. 28847215

[hbm70580-bib-0017] D'Esposito, M. , B. R. Postle , J. Jonides , and E. E. Smith . 1999. “The Neural Substrate and Temporal Dynamics of Interference Effects in Working Memory as Revealed by Event‐Related Functional MRI.” Proceedings of the National Academy of Sciences of the United States of America 96, no. 13: 7514–7519. 10377446 10.1073/pnas.96.13.7514 PMC22117

[hbm70580-bib-0018] Faulkner, J. W. , D. L. Snell , D. Shepherd , and A. Theadom . 2021. “Turning Away From Sound: The Role of Fear Avoidance in Noise Sensitivity Following Mild Traumatic Brain Injury.” Journal of Psychosomatic Research 151: 110664. 10.1016/j.jpsychores.2021.110664. 34749069

[hbm70580-bib-0019] Glover, G. H. 1999. “Deconvolution of Impulse Response in Event‐Related BOLD fMRI.” NeuroImage 9, no. 4: 416–429. 10191170 10.1006/nimg.1998.0419

[hbm70580-bib-0064] Hall, C. N. , C. Howarth , Z. Kurth‐Nelson , and A. Mishra . 2016. “Interpreting BOLD: Towards a Dialogue Between Cognitive and Cellular Neuroscience.” Philosophical Transactions of the Royal Society of London. Series B, Biological Sciences 371, no. 1705. 10.1098/rstb.2015.0348 PMC5003850 27574302

[hbm70580-bib-0020] Hammeke, T. A. , M. McCrea , S. M. Coats , et al. 2013. “Acute and Subacute Changes in Neural Activation During the Recovery From Sport‐Related Concussion.” Journal of the International Neuropsychological Society 19, no. 8: 863–872. 10.1017/S1355617713000702. 23829951

[hbm70580-bib-0065] Havsteen, I. , A. Ohlhues , K. H. Madsen , J. D. Nybing , H. Christensen , and A. Christensen . 2017. “Are Movement Artifacts in Magnetic Resonance Imaging a Real Problem?‐A Narrative Review.” Frontiers in Neurology 8: 232. 28611728 10.3389/fneur.2017.00232 PMC5447676

[hbm70580-bib-0021] Hergert, D. C. , V. Sicard , D. D. Stephenson , et al. 2022. “Test‐Retest Reliability of a Semi‐Structured Interview to Aid in Pediatric Traumatic Brain Injury Diagnosis.” Journal of the International Neuropsychological Society 28, no. 7: 687–699. 10.1017/S1355617721000928. 34376268 PMC8831656

[hbm70580-bib-0022] Hoover, E. C. , P. E. Souza , and F. J. Gallun . 2017. “Auditory and Cognitive Factors Associated With Speech‐In‐Noise Complaints Following Mild Traumatic Brain Injury.” Journal of the American Academy of Audiology 28, no. 4: 325–339. 10.3766/jaaa.16051. 28418327 PMC5600820

[hbm70580-bib-0023] Hou, R. H. , R. Moss‐Morris , R. Peveler , K. Mogg , B. P. Bradley , and A. Belli . 2012. “When a Minor Head Injury Results in Enduring Symptoms: A Prospective Investigation of Risk Factors for Postconcussional Syndrome After Mild Traumatic Brain Injury.” Journal of Neurology, Neurosurgery, and Psychiatry 83, no. 2: 217–223. 10.1136/jnnp-2011-300767. 22028384

[hbm70580-bib-0024] Howell, D. R. , L. R. Osternig , M. C. Koester , and L. S. Chou . 2014. “The Effect of Cognitive Task Complexity on Gait Stability in Adolescents Following Concussion.” Experimental Brain Research 232, no. 6: 1773–1782. 10.1007/s00221-014-3869-1. 24531643

[hbm70580-bib-0025] Hylin, M. J. , A. L. Kerr , and R. Holden . 2017. “Understanding the Mechanisms of Recovery and/or Compensation Following Injury.” Neural Plasticity 2017: 7125057. 10.1155/2017/7125057. 28512585 PMC5415868

[hbm70580-bib-0026] Irlbacher, K. , A. Kraft , S. Kehrer , and S. A. Brandt . 2014. “Mechanisms and Neuronal Networks Involved in Reactive and Proactive Cognitive Control of Interference in Working Memory.” Neuroscience and Biobehavioral Reviews 46, no. Pt 1: 58–70. 10.1016/j.neubiorev.2014.06.014. 25003803

[hbm70580-bib-0027] Johnson, V. E. , J. E. Stewart , F. D. Begbie , J. Q. Trojanowski , D. H. Smith , and W. Stewart . 2013. “Inflammation and White Matter Degeneration Persist for Years After a Single Traumatic Brain Injury.” Brain 136, no. Pt 1: 28–42. 10.1093/brain/aws322. 23365092 PMC3562078

[hbm70580-bib-0028] Jonsson, C. A. , G. Horneman , and I. Emanuelson . 2004. “Neuropsychological Progress During 14 Years After Severe Traumatic Brain Injury in Childhood and Adolescence.” Brain Injury 18, no. 9: 921–934. 10.1080/02699050410001671900. 15223744

[hbm70580-bib-0029] Larson, M. J. , P. E. Clayson , and T. J. Farrer . 2012. “Performance Monitoring and Cognitive Control in Individuals With Mild Traumatic Brain Injury.” Journal of the International Neuropsychological Society 18, no. 2: 323–333. 10.1017/S1355617711001779. 22272692

[hbm70580-bib-0030] Larson, M. J. , T. J. Farrer , and P. E. Clayson . 2011. “Cognitive Control in Mild Traumatic Brain Injury: Conflict Monitoring and Conflict Adaptation.” International Journal of Psychophysiology 82, no. 1: 69–78. 21392543 10.1016/j.ijpsycho.2011.02.018

[hbm70580-bib-0031] Luna, B. 2009. “Developmental Changes in Cognitive Control Through Adolescence.” Advances in Child Development and Behavior 37: 233–278. 19673164 10.1016/s0065-2407(09)03706-9 PMC2782527

[hbm70580-bib-0032] Maki‐Marttunen, V. , T. Hagen , and T. Espeseth . 2019. “Proactive and Reactive Modes of Cognitive Control Can Operate Independently and Simultaneously.” Acta Psychologica 199: 102891. 10.1016/j.actpsy.2019.102891. 31400651

[hbm70580-bib-0033] Mayer, A. R. , D. M. Cohen , C. J. Wertz , et al. 2020. “Radiologic Common Data Elements Rates in Pediatric Mild Traumatic Brain Injury.” Neurology 94, no. 3: e241–e253. 10.1212/WNL.0000000000008488. 31645467 PMC7108809

[hbm70580-bib-0034] Mayer, A. R. , F. M. Hanlon , and J. Ling . 2015. “Gray Matter Abnormalities in Pediatric Mild Traumatic Brain Injury.” Journal of Neurotrauma 32, no. 10: 723–730. 10.1089/neu.2014.3534. 25313896

[hbm70580-bib-0035] Mayer, A. R. , T. B. Meier , J. M. Ling , et al. 2023. “Increased Brain Age and Relationships With Blood‐Based Biomarkers Following Concussion in Younger Populations.” Journal of Neurology 270, no. 12: 5835–5848. 10.1007/s00415-023-11931-8. 37594499 PMC10632216

[hbm70580-bib-0036] Mayer, A. R. , S. G. Ryman , F. M. Hanlon , A. B. Dodd , and J. M. Ling . 2017. “Look Hear! The Prefrontal Cortex Is Stratified by Modality of Sensory Input During Multisensory Cognitive Control.” Cerebral Cortex 27, no. 5: 2831–2840. 27166168 10.1093/cercor/bhw131 PMC6059096

[hbm70580-bib-0037] Mayer, A. R. , D. D. Stephenson , A. B. Dodd , et al. 2020. “Comparison of Methods for Classifying Persistent Post‐Concussive Symptoms in Children.” Journal of Neurotrauma 37, no. 13: 1504–1511. 10.1089/neu.2019.6805. 31964232 PMC7307699

[hbm70580-bib-0038] Mayer, A. R. , T. Toulouse , S. Klimaj , J. Ling , A. Pena , and P. Bellgowan . 2014. “Investigating the Properties of the Hemodynamic Response Function Following Mild Traumatic Brain Injury.” Journal of Neurotrauma 31, no. 2: 189–197. 10.1089/neu.2013.3069. 23965000 PMC3900017

[hbm70580-bib-0039] McAllister, T. W. , M. B. Sparling , L. A. Flashman , S. J. Guerin , A. C. Mamourian , and A. J. Saykin . 2001. “Differential Working Memory Load Effects After Mild Traumatic Brain Injury.” NeuroImage 14, no. 5: 1004–1012. 11697932 10.1006/nimg.2001.0899

[hbm70580-bib-0040] McInnes, K. , C. L. Friesen , D. E. MacKenzie , D. A. Westwood , and S. G. Boe . 2017. “Mild Traumatic Brain Injury (mTBI) and Chronic Cognitive Impairment: A Scoping Review.” 12, no. 4: e0174847. 10.1371/journal.pone.0174847. PMC5388340 28399158

[hbm70580-bib-0041] Miller, N. R. , A. L. Yasen , L. F. Maynard , L. S. Chou , D. R. Howell , and A. D. Christie . 2014. “Acute and Longitudinal Changes in Motor Cortex Function Following Mild Traumatic Brain Injury.” Brain Injury 28, no. 10: 1270–1276. 10.3109/02699052.2014.915987. 24841536

[hbm70580-bib-0042] Moore, R. D. , C. H. Hillman , and S. P. Broglio . 2014. “The Persistent Influence of Concussive Injuries on Cognitive Control and Neuroelectric Function.” Journal of Athletic Training 49, no. 1: 24–35. 10.4085/1062-6050-49.1.01. 24377962 PMC3917292

[hbm70580-bib-0043] Muscara, F. , C. Catroppa , and V. Anderson . 2008. “The Impact of Injury Severity on Executive Function 7‐10 Years Following Pediatric Traumatic Brain Injury.” Developmental Neuropsychology 33, no. 5: 623–636. http://www.ncbi.nlm.nih.gov/entrez/query.fcgi?cmd=Retrieve&db=PubMed&dopt=Citation&list_uids=18788014. 18788014 10.1080/87565640802171162

[hbm70580-bib-0044] Nathaniel, U. , T. V. Wick , J. L. Ling , et al. 2025. “Regional and Global Changes in Brain Structure 1‐Year Post Pediatric “Mild” Traumatic Brain Injury.” Annals of Neurology 98, no. 2: 341–353. 10.1002/ana.27259. 40476699 PMC12235941

[hbm70580-bib-0045] Pardini, J. E. , D. A. Pardini , J. T. Becker , et al. 2010. “Postconcussive Symptoms Are Associated With Compensatory Cortical Recruitment During a Working Memory Task.” Neurosurgery 67, no. 4: 1020–1027. 10.1227/NEU.0b013e3181ee33e2. 20881565 PMC2998066

[hbm70580-bib-0046] Raichle, M. E. 2015. “The Brain's Default Mode Network.” Annual Review of Neuroscience 38: 433–447. 10.1146/annurev-neuro-071013-014030. 25938726

[hbm70580-bib-0047] Ryman, S. G. , A. A. El Shaikh , N. A. Shaff , et al. 2019. “Proactive and Reactive Cognitive Control Rely on Flexible Use of the Ventrolateral Prefrontal Cortex.” Human Brain Mapping 40, no. 3: 955–966. 10.1002/hbm.24424. 30407681 PMC6865444

[hbm70580-bib-0048] Seeger, T. A. , A. Kirton , M. J. Esser , et al. 2017. “Cortical Excitability After Pediatric Mild Traumatic Brain Injury.” Brain Stimulation 10, no. 2: 305–314. 10.1016/j.brs.2016.11.011. 27916406

[hbm70580-bib-0049] Seeley, W. W. , V. Menon , A. F. Schatzberg , et al. 2007. “Dissociable Intrinsic Connectivity Networks for Salience Processing and Executive Control.” Journal of Neuroscience 27, no. 9: 2349–2356. 17329432 10.1523/JNEUROSCI.5587-06.2007 PMC2680293

[hbm70580-bib-0050] Shenhav, A. , M. M. Botvinick , and J. D. Cohen . 2013. “The Expected Value of Control: An Integrative Theory of Anterior Cingulate Cortex Function.” Neuron 79, no. 2: 217–240. 10.1016/j.neuron.2013.07.007. 23889930 PMC3767969

[hbm70580-bib-0051] Sicard, V. , D. D. Stephenson , A. B. Dodd , et al. 2021. “Is the Prefrontal Cortex Organized by Supramodal or Modality‐Specific Sensory Demands During Adolescence?” Developmental Cognitive Neuroscience 51: 101006. 34419765 10.1016/j.dcn.2021.101006 PMC8379626

[hbm70580-bib-0052] Silverberg, N. D. , G. L. Iverson , ACRM Brain Injury Special Interest Group Mild TBI Task Force members , et al. 2023. “The American Congress of Rehabilitation Medicine Diagnostic Criteria for Mild Traumatic Brain Injury.” Archives of Physical Medicine and Rehabilitation 104, no. 8: 1343–1355. 10.1016/j.apmr.2023.03.036. 37211140

[hbm70580-bib-0053] Smith, S. M. , M. Jenkinson , M. W. Woolrich , et al. 2004. “Advances in Functional and Structural MR Image Analysis and Implementation as FSL.” NeuroImage 23: S208–S219. 15501092 10.1016/j.neuroimage.2004.07.051

[hbm70580-bib-0054] Sylvester, C. M. , M. Corbetta , M. E. Raichle , et al. 2012. “Functional Network Dysfunction in Anxiety and Anxiety Disorders.” Trends in Neurosciences 35, no. 9: 527–535. 10.1016/j.tins.2012.04.012. 22658924 PMC3432139

[hbm70580-bib-0055] Talavage, T. M. , E. Nauman , E. L. Breedlove , et al. 2014. “Functionally‐Detected Cognitive Impairment in High School Football Players Without Clinically‐Diagnosed Concussion.” Journal of Neurotrauma 31, no. 4: 327–338. http://www.ncbi.nlm.nih.gov/entrez/query.fcgi?cmd=Retrieve&db=PubMed&dopt=Citation&list_uids=20883154. 20883154 10.1089/neu.2010.1512 PMC3922228

[hbm70580-bib-0056] Taylor, C. A. , J. M. Bell , M. J. Breiding , and L. Xu . 2017. “Traumatic Brain Injury‐Related Emergency Department Visits, Hospitalizations, and Deaths ‐ United States, 2007 and 2013.” MMWR Surveillance Summaries 66, no. 9: 1–16. 10.15585/mmwr.ss6609a1. PMC5829835 28301451

[hbm70580-bib-0057] Tenório, R. B. , A. A. Vieira , G. L. Franklin , M. V. Della Coletta , H. A. G. Teive , and C. H. F. Camargo . 2025. “The Cognitive Cerebellum: A Hub for Motor, Emotional, and Executive Control.” Arquivos de Neuro‐Psiquiatria 83, no. 12: 1–12. 10.1055/s-0045-1814378. PMC12832161 41581509

[hbm70580-bib-0058] Tonks, J. , W. H. Williams , P. Yates , and A. Slater . 2011. “Cognitive Correlates of Psychosocial Outcome Following Traumatic Brain Injury in Early Childhood: Comparisons Between Groups of Children Aged Under and Over 10 Years of Age.” Clinical Child Psychology and Psychiatry 16, no. 2: 185–194. 10.1177/1359104511403583. 21571762

[hbm70580-bib-0059] Urban, K. , L. Schudlo , M. Keightley , S. Alain , N. Reed , and T. Chau . 2021. “Altered Brain Activation in Youth Following Concussion: Using a Dual‐Task Paradigm.” Developmental Neurorehabilitation 24, no. 3: 187–198. 10.1080/17518423.2020.1825539. 33012188

[hbm70580-bib-0060] van der Horn, H. J. , A. B. Dodd , T. V. Wick , et al. 2023. “Neural Correlates of Cognitive Control Deficits in Pediatric Mild Traumatic Brain Injury.” Human Brain Mapping 44, no. 17: 6173–6184. 10.1002/hbm.26504. 37800467 PMC10619369

[hbm70580-bib-0061] Wille, C. , and M. Ebersbach . 2016. “Semantic Congruency and the (Reversed) Colavita Effect in Children and Adults.” Journal of Experimental Child Psychology 141: 23–33. 10.1016/j.jecp.2015.07.015. 26311396

[hbm70580-bib-0062] Zandbelt, B. B. , M. Bloemendaal , S. F. Neggers , R. S. Kahn , and M. Vink . 2013. “Expectations and Violations: Delineating the Neural Network of Proactive Inhibitory Control.” Human Brain Mapping 34, no. 9: 2015–2024. 10.1002/hbm.22047. 22359406 PMC6869973

[hbm70580-bib-0063] Zhao, P. , X. Y. Wang , Q. Wang , et al. 2023. “Altered Fractional Amplitude of Low‐Frequency Fluctuations in the Superior Temporal Gyrus: A Resting‐State fMRI Study in Anxious Depression.” BMC Psychiatry 23, no. 1: 847. 10.1186/s12888-023-05364-w. 37974113 PMC10655435

