# Supplementary material for: Injury Severity Influences Long‐Term Cognitive Control in Pediatric “Mild” Traumatic Brain Injury
Source: Hum Brain Mapp. 2026 Jul 3;47(10):e70580. doi: 10.1002/hbm.70580 (PMC13331867; doi:10.1002/hbm.70580)
Supplement: Supplementary file 1 — Figure S1: Participant recruitment and retention. Figure S2: Congruency effects in whole‐brain voxel‐wise analysis. Table S1: Primary and secondary clinical and cognitive measures. Table S2: Significant regions of activation for the Group × Visit × Phase interaction during the Attend‐Auditory condition of the multimodal attention task. Table S3: Significant regions of activation for the Group × Phase × Congruency interaction during the Attend‐Auditory condition of the multimodal attention task. Table S4: Significant regions of activation for the Group × Visit × Congruency interaction during the Attend‐Auditory condition of the multimodal attention task. [file HBM-47-e70580-s001.docx]

**Supplemental Materials**

1. **Methods**
   1. Participants

A total of 279 patients with pediatric “mild” traumatic injury (pmTBI), along with 242 health controls (HC) were recruited at V1 and passed clinical exclusions (listed below, see Figure S1). The final sample at each visit was determined based on those who completed the fMRI task, passed quality assurance checks (QC), were not an outlier based on motion (mean framewise displacement >3*interquartile range), behavioral performance (accuracy <=0.399 on two or more conditions of the four behavioral conditions based on a binomial distribution; reaction time >3*interquartile range) or voxel-wise activations (3 standard deviations above the group mean on five or more of the eight imaging conditions). This resulted in the exclusion of 28 participants (20 pmTBI) that did not complete the fMRI task, 5 participants (1 pmTBI) were excluded based on QC, 25 participants (15 pmTBI) were motion outliers, 3 participants (1 pmTBI) were behavioral outliers, and 12 participants (6 pmTBI) were voxel-wise outliers. This resulted in a final sample of 236 pmTBI and 212 HC at Visit[V]1 (Figure S1). Participants with incomplete data or those excluded during QC or outlier checks were still eligible for subsequent visit analyses, provided they met other exclusionary criteria and passed quality assurance.

Attrition occurred for 47 pmTBI (83.2% retention) and 14 HC (94.2% retention) between V1 and V2. One pmTBI was unable to complete the study due to COVID-19 pandemic restrictions. This resulted in 231 pmTBI and 228 HC eligible to participate at V2. Based on clinical exclusions, 3 participants (1 pmTBI) were excluded due to a positive drug screen, 8 participants (7 pmTBI) due to a scanner upgrade, 2 HC were diagnosed with a new psychiatric disorder between V1 and V2, 3 HC were disenrolled from the study due to unreliable arrival at V1, 3 HC had new MRI contraindications, and 6 participants (5 pmTBI) were excluded due to new braces. An additional 5 pmTBI participants were unable to return for V2 only but returned for V3. Following clinical exclusions, 26 participants (20 pmTBI) did not complete the fMRI task, 2 HC were excluded following QC, 13 participants (8 pmTBI) were excluded as motion outliers, 1 HC was excluded as a behavioral outlier and 26 participants (14 pmTBI) were voxel-wise outliers. This resulted in 171 pmTBI and 190 HC eligible for analyses at V2.

Retention rates for V3 included 70.6% of the recruited pmTBI and 83.1% of the recruited HC prior to exclusions at V3. Three pmTBI participants were further lost to follow-up due to COVID-19 pandemic restrictions. This resulted in 194 pmTBI and 201 HC eligible at V3. Four participants (1 pmTBI) had a positive drug screen, 37 participants (11 pmTBI) were excluded due to a scanner upgrade, 2 pmTBI had a new medical condition, and 5 HC were diagnosed with a new psychiatric disorder between V2 and V3. An additional 12 participants (6 pmTBI) could not complete scan due to new braces, 25 participants (18 pmTBI) did not complete the fMRI task, 3 pmTBI were excluded following QC, 6 participants (5 pmTBI) were excluded due to excessive motion and 10 participants (4 pmTBI) were excluded following voxel-wise outlier analysis. This resulted in 144 pmTBI and 147 HC for final analyses at V3.

- 1. Common Data Element Measures

A battery of clinical and neuropsychological measures was administered to pmTBI and HC at all three visits. Clinical measures included medical history, the New Mexico Assessment of Pediatric Traumatic Brain Injury semi-structured interview (Hergert et al., 2022) self-report of Tanner stage of development (Kriz et al., 2016) and the Glasgow Outcome Scale Extended (GOS-E) Pediatric Revision (Beers et al., 2012). Additional measures included the Alcohol, Smoking and Substance Involvement Screening Test (ASSIST; Group, 2002), self and parent reports of concussion symptom severity for retrospective and current periods including Post-Concussive Symptom Inventory (PCSI; Gioia et al., 2008; Gioia et al., 2009), Conflict and Behavioral Questionnaire (CBQ; Prinz et al., 1979), and Pediatric Quality of Life Inventory (PedsQL - Generic Core; Varni et al., 1999). With the author’s permission, the PCSI was adapted to be administered to 12 year old participants using the 13-18 year-old version, and the 5-12 year-old version was used for participants under 12. Additional revisions included more clearly specified retrospective (i.e., one month prior to initial visit) and V1 (day of initial visit) reporting instructions. All PCSI summary scores were normalized to a percentage value (sum of individual rating/maximum score * 100) to account for differences in scale ratings for specific age ranges.

Other measures independently completed by participants included current and retrospective Patient Reported Outcomes Measurement Information System (PROMIS) for sleep (Forrest et al., 2018), anxiety, and depression (Irwin et al., 2010), a brief pain rating (0-10 Likert scale; Farrar et al., 2001), and Headache Impact Test (HIT; Genizi et al., 2025). Parents alone filled out the Strengths and Difficulties (SDQ; Goodman, 1997) and parental distress was measured with the Brief Symptom Inventory Questionnaire (BSI-18; Derogatis & Fitzpatrick, 2004). A urine screen for amphetamines, methamphetamines, benzodiazepines, barbiturates, cocaine, marijuana, methadone, opiates, phencyclidine, and Methylenedioxy-methamphetamine use was administered to all participants, with a positive screen resulting in exclusion from the study, except for recreational marijuana use.

The cognitive battery included paper-and-pencil tests of premorbid cognitive ability (Wide Range Achievement Test [WRAT]; Wilkinson & Robertson, 2006), a shortened measure of effort (Test of Memory Malingering [TOMMe10]; Denning, 2012), and selected tests from the Delis-Kaplan Executive Function System (DKEFS; Delis et al., 2001), the Hopkins Verbal Learning Test-Revised (HVLT), and the Wechsler Intelligence Scales depending on initial age at assessment. Specifically, the Wechsler Adult Intelligence Scale-IV (WAIS-IV; Wechsler, 2008) was used for participants 16-18 years old at enrollment whereas the Wechsler Intelligence Scale for Children-V (WISC-V; Wechsler, 2014) was used for participants 8-15 years old at enrollment. Composite measures of attention (DKEFS color-word interference conditions 1-3), processing speed (WAIS-IV/WISC-V digit symbol coding and symbol search), working memory (WISC-V/WAIS-IV digit span backwards trial), executive function (DKEFS trail making test condition-4, verbal fluency, color-word interference condition 4) and long-term memory recall (HVLT Delay) were compiled to create specific cognitive domains by averaging t-scores from individual tests.

- 1. MR Imaging Parameters

Participants were scanned using either 3T Siemens TrioTim or a PRISMA fit MRI scanner with a 32-channel head coil. The TrioTim protocol included a high-resolution 5-echo Magnetization Prepared Rapid Acquisition Gradient Echo (MPRAGE) T_1_–weighted [repetition time (TR)=2530 ms; echo times (TE)=1.64, 3.5, 5.36, 7.22, 9.08 ms; inversion time (TI)=1200 ms; flip angle=7°; number of excitations (NEX)=1; slice thickness=1 mm; field of view (FOV)=256 mm; matrix size=256 × 256; isotropic voxels=1 mm3], a T_2_-weighted sequence [TR=15500 ms; TE=77 ms; flip angle=155°; NEX=1; slice thickness=1.5mm; FOV=220 mm; matrix size=192×192; voxel size=1.15 × 1.1.5 × 1.5 mm], a susceptibility-weighted (SWI) sequence [TR = 28 ms; TE = 20.0 ms; flip angle = 15°; NEX = 1; slice thickness = 1.5 mm; FOV = 192 × 256; matrix size = 192 × 256; voxel size = 1.00 × 1.00 × 1.50 mm], and a fluid-attenuated inversion recovery (FLAIR) sequence [TR = 10380 ms; TE = 88.0 ms; TI = 2500 ms; flip angle = 140; NEX = 1; slice thickness = 3 mm; FOV = 256; matrix size = 320×320; 50 interleaved slices; 0.80×0.80×3.00 mm voxels].

Although identical base sequences were deployed, minor modifications in sequence parameters were made during the upgrade to the new scanner. The modified sequences on the PRISMA fit scanner included a high-resolution 5-echo Magnetization Prepared Rapid Acquisition Gradient Echo (MPRAGE) T_1_–weighted [repetition time (TR)=2530 ms; echo times (TE)=1.61, 3.47, 5.33, 7.19, 9.05 ms; inversion time (TI)=1200 ms; flip angle=7°; number of excitations (NEX)=1; slice thickness=1 mm; field of view (FOV)=256 mm; matrix size=256 × 256; isotropic voxels=1 mm3], a T_2_-weighted sequence [TR=3200 ms; TE=428 ms; flip angle=120°; NEX=1; slice thickness=1mm; FOV=256 mm; matrix size=256 × 256; voxel size=1.0 × 1.0 × 1.0 mm], a FLAIR sequence [TR=10380 ms; TE=89 ms; TI=2500 ms; flip angle=160°; NEX=1; slice thickness=3mm; FOV=256 mm; matrix size=320 × 320; 50 interleaved slices; voxel size=0.8 × 0.8 × 3 mm], and a SWI sequence [TR = 28 ms; TE = 20.0 ms; flip angle = 15°; NEX = 1; slice thickness = 1.5 mm; FOV = 192 × 256; matrix size = 192 × 256; 88 interleaved slices; voxel size = 1.00 × 1.00 × 1.50 mm].

- 1. Clinical analyses

Bonferroni corrections were applied to all clinical and cognitive results, based on *a priori* primary and secondary measure designation when the initial grant was proposed (Supplementary Table S1). Three domains (PCS burden, pediatric quality of life, and self-reported behavioral disturbances) were included in the primary clinical measures with a Bonferroni correction of p<0.0167 (p=0.05/3). Seven domains (pain scale, headache, sleep, anxiety, depression, global outcome, and behavioral and emotional difficulties) were included in the secondary clinical measures with a Bonferroni correction of p<0.007 (p=0.05/7). Two domains (attention and processing speed) were included in the primary cognitive measures with a Bonferroni correction of *p*<0.025 (p=0.05/2). Three domains (executive function, long-term memory, and working memory) were included in the secondary cognitive measures with a Bonferroni correction of *p*<0.0167 (p=0.05/3). Normative scores for children do not exist for the HVLT; therefore, age was included as a covariate only for the long-term memory domain.

1. **Supplemental Results**
   1. Clinical outcomes

Primary clinical measures revealed a significant Group×Age interaction (Wald-*χ*^2^=6.16; *p*=0.013) for self-reported PCS severity. Follow-up tests revealed a significant negative association between age and PCS severity for HC (Wald-*χ*^2^=12.79; β=−0.043; *p*=3.5e-04), and a non-significant association for pmTBI (β=0.017; *p*>0.05). There was a significant Group×Visit×Age interaction (Wald-*χ*^2^=11.61, *p*=0.003) for self-reported behavioural disturbances. However, follow-up analyses did not reveal a significant Group×Age interaction at any of the visits (*p*’s > Bonferroni corrected α=0.0167).

Results for secondary clinical measures indicated a significant main effect of Age (Wald-*χ*^2^=7.23, *p*=0.007) for sleep disturbance. There was a significant Group×Visit×Age interaction (Wald-*χ*^2^=11.52, *p*=0.003) for anxiety. However, follow-up analyses did not reveal a significant Group×Age interaction at any of the visits (all *p*’s < Bonferroni corrected α=0.007).

Results for secondary neuropsychological domains indicated a significant Group×Age interaction (Wald-*χ*^2^=7.95, *p*=0.005) for long-term memory. However, follow-up analyses indicated that there was a significant relationship between age and long-term memory recall that was positive for both HC (β=0.034; *p*<0e-36) and pmTBI (β=0.015; *p*=0.007). The main effect of Visit was also significant for long-term memory (Wald-*χ*^2^=44.38, *p*=2.3e-10) with increased performance at V3 relative to V1 (*p*=2.8e-07) and V2 (*p*=1.9e-10), and no difference in performance at V2 relative to V1 (*p*>0.05). There was a significant main effect of Visit for executive function (Wald-*χ*^2^=277.46, *p*<0e-36), which indicated an improvement in performance from V1 to V2 to V3 (all *p*’s<0e-36).

- 1. Behavioral task performance

For accuracy, a significant effect of Congruency (congruent>incongruent) was found for both Attend-Auditory (Wald-*χ*^2^=101.35, *p*<0e-36) and Attend-Visual conditions (Wald-χ^2^ =545.16, *p*<0e-36). There was also a main effect of Visit for the Attend-Visual conditions (Wald-*χ*^2^=37.25, *p*=8.2e-09), with lower accuracy at V1 relative to V2 (*p*=3.2e-07) and V3 (*p*=3.2e-09), and no difference between V2 and V3. For reaction time, a significant effect of Congruency (congruent<incongruent) was found for both Attend-Auditory (Wald-*χ*^2^=440.08, *p*<0e-36) and Attend-Visual conditions (Wald-*χ*^2^=930.89, *p*<0e-36). A main effect of Visit was also found for both Attend-Auditory (Wald-*χ*^2^=13.39, *p*=0.001) and Attend-Visual conditions (Wald-*χ*^2^=11.89, *p*=0.003), with slower RTs at V1 relative to V2 and V3 (all *p*’s<0.006), and no difference between V2 and V3.

- 1. Whole-brain group fMRI analyses

The Attend-Auditory condition showed a main effect of Congruency (see Supplemental figure S2a) with greater activation for congruent relative to incongruent trials in left insula and greater deactivation for congruent relative to incongruent trials in left inferior temporal gyrus and left cingulate gyrus, there was also a pattern of greater activation for incongruent>congruent in right middle frontal gyrus and greater deactivation (incongruent>congruent) in right postcentral and precentral gyri, bilateral middle temporal gyrus and right insula. There was also a main effect of Phase with greater activation during late peak>peak phase in right cerebellum and right orbital gyrus, with additional clusters in left cerebellum, left middle frontal gyrus and bilateral middle temporal gyrus indicating positive activation during late peak relative to deactivation during the peak phase. Other clusters indicated greater deactivation for late peak relative to peak phase in right parahippocampal gyrus, right precentral gyrus and left precuneus, with right middle occipital gyrus showing greater deactivation during late peak relative to peak phase.

The Attend-Visual condition also showed a main effect of Congruency (see Supplemental figure S2b) with greater activation for incongruent>congruent trials in bilateral premotor cortex, bilateral precentral gyrus, right superior frontal gyrus, right middle temporal gyrus, left superior temporal gyrus, left middle frontal gyrus; and greater activation for congruent vs. incongruent in right angular gyrus. A main effect of Phase (late peak>peak) was significant in several clusters in the right hemisphere including right middle and superior frontal gyrus, right premotor cortex, right middle and superior temporal gyrus, right cingulum and right cerebellum, with bilateral putamen indicating greater activation for peak relative to late peak phase. There was also a main effect of Visit (V1>V3>V2) in left middle occipital gyrus, right cingulate gyrus and right thalamus, as well as in right superior temporal gyrus (V1>V2>V3).

**Figures**

**
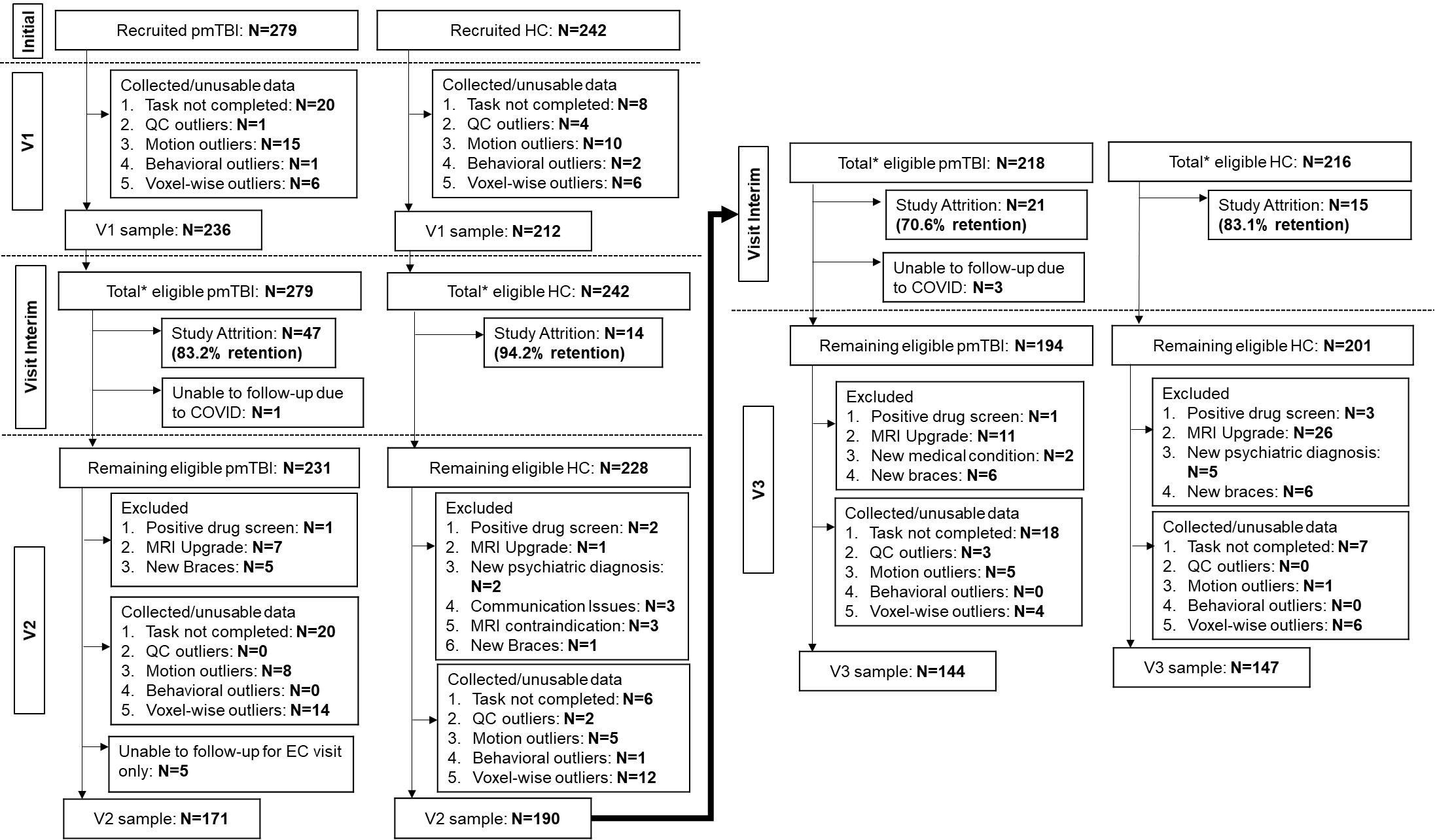
**

**Figure S1.** Participant recruitment and retention. Flowchart of enrolment, inclusion and data quality control from visits 1 (V1), 2 (V2), and 3 (V3) for patients with pediatric “mild” traumatic brain injury (pmTBI) and matched healthy controls (HC). The asterisk denotes the total number of participants who were eligible to return, which is a sum of participants with usable clinical and magnetic resonance imaging (MRI) data and those whose data was excluded at previous visits because of quality assurance issues.


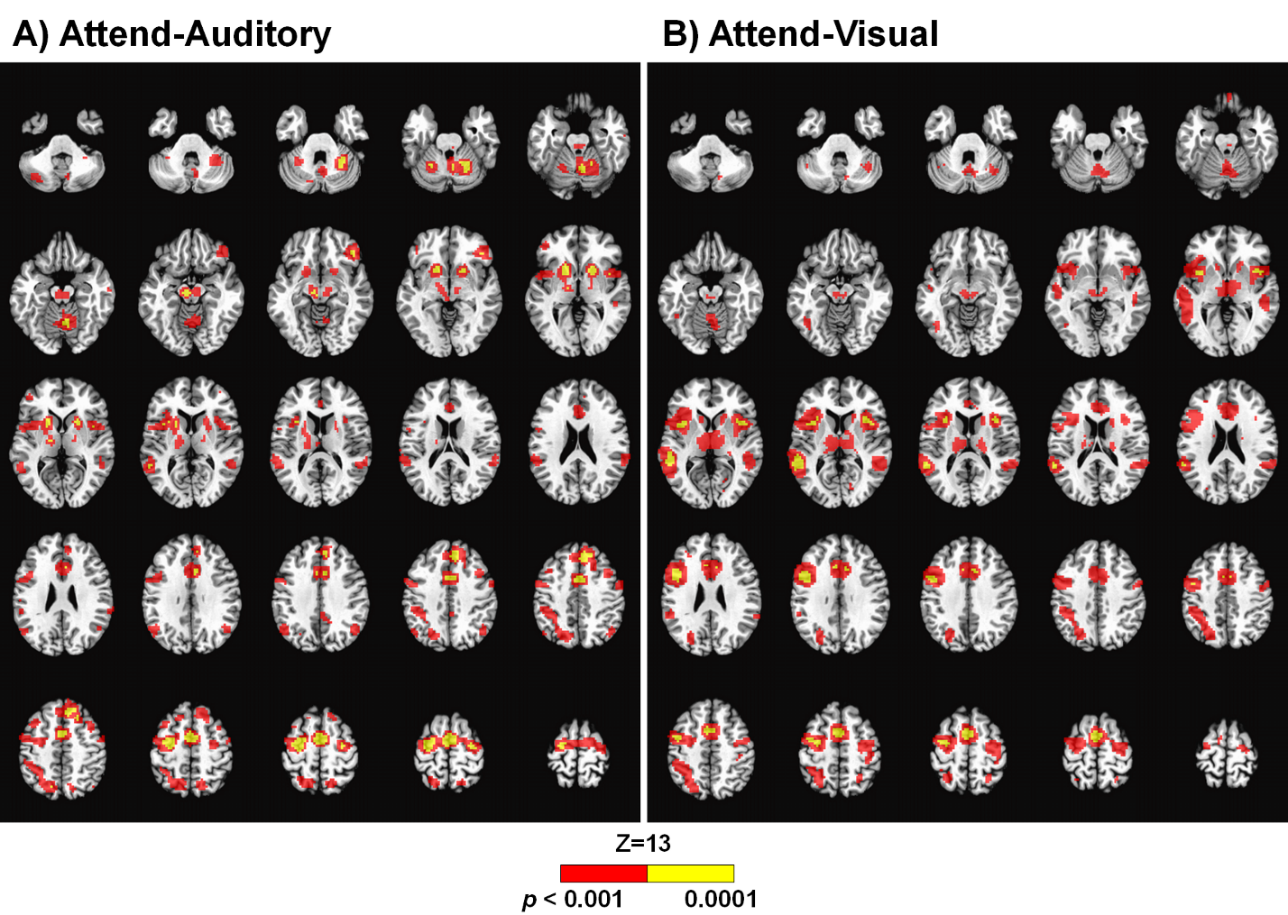


**Figure S2.** Congruency effects in whole-brain voxel-wise analysis. Regions exhibiting significant effects associated with congruency in the auditory (panel **A**) and visual (panel **B**) modalities. Location of the axial (Z) slice is given according to the Talairach atlas.

**Tables**

**Table S1.** Primary and secondary clinical and cognitive measures.

| **Instrument** | **Measured domain** | **Status** | **Rater** | **Visit** |
| --- | --- | --- | --- | --- |
| **Demographics** | | | | |
| NewMAP TBI | Self-reported TBI history | Secondary | C & P | R, V1, V2, V3 |
| Tanner Stage of Development | Pubertal development | Secondary | C | V1, V2 & V3 |
| ASSIST | Use of alcohol and other drugs | Secondary | C | V1, V2 & V3 |
| BSI-18 | Parental psychopathology | Secondary | P | V1, V2 & V3 |
| **Clinical Domain** | | | | |
| PCSI | Post-concussive symptoms | Primary | C | R, V1, V2 & V3 |
| PROMIS Sleep | Sleep disturbance | Secondary | C | R, V1, V2 & V3 |
| PROMIS Anxiety | Anxiety symptoms | Secondary | C | R, V1, V2 & V3 |
| PROMIS Depression | Depressive symptoms | Secondary | C | R, V1, V2 & V3 |
| Pain scale | Pain | Secondary | C | R, V1, V2 & V3 |
| HIT-6 | Headache symptoms | Secondary | C | R, V1, V2 & V3 |
| CBQ | Family conflict | Primary | C | R, V1, V2 & V3 |
| SDQ | Behavioral screening for psychological attributes | Secondary | P | R, V2 & V3 |
| PedsQL | Health-related quality of life | Primary | C | R & V2 & V3 |
| GOS-E | Functional outcome | Secondary | C & P | V1, V2 & V3 |
| **Cognitive Domain** | | | | |
| TOMMe10 | Measure of effort | Secondary | C | V1, V2 & V3 |
| WRAT-4 | Premorbid reading ability | Secondary | C | V1, V2 & V3 |
| DKEFS Color-Word interference Cond 1-3 | Attention | Primary | C | V1, V2 & V3 |
| WAIS-IV/WISC-V Coding and Symbol Search | Processing speed | Primary | C | V1, V2 & V3 |
| WISC-V/WAIS-IV Digit Span Backwards | Working memory | Secondary | C | V1, V2 & V3 |
| DKEFS Trail Making Test Conditions 2 & 4, Verbal Fluency, Color-Word interference Cond 4 | Executive function | Secondary | C | V1, V2 & V3 |
| HVLT Delayed Recall | Long-Term Memory Recall | Secondary | C | V1, V2 & V3 |

Notes: Instrument—NewMAP TBI=New Mexico Assessment of Pediatric Traumatic Brain Injury, ASSIST=The Alcohol, Smoking and Substance Involvement Screening Test, BSI=Brief Symptom Inventory-18, PCSI=Post-Concussion Symptom Inventory, PROMIS=Patient-Reported Outcomes Measurement Information System, HIT-6=Headache Impact Test, CBQ=Conflict Behavior Questionnaire, SDQ=Strengths and Difficulties Questionnaire, PedsQL=Pediatric Quality of Life Inventory, GOS-E=Glasgow Outcome Scale Extended, TOMMe10=Test of Memory Malingering, WRAT-4=Wide Range Achievement Test, DKEFS=Delis-Kaplan Executive Function System, WAIS-IV=Wechsler Adult Intelligence Scale–IV, WISC-V=Wechsler Intelligence Scale for Children–V, HVLT=Hopkins Verbal Learning Test; Rater—C=child, P=parent; Visit—R=retrospective, Visit—V1=~1-week post-injury,V2=~4 months post-injury; V3=~1-year post-injury.

**Table S2.** Significant regions of activation for the Group×Visit×Phase interaction during the Attend-Auditory condition of the multimodal attention task.

| **Region** | **Laterality** | **CM**  **(x,y,z)** | **Volume (µL)** |
| --- | --- | --- | --- |
| Brodmann Areas 21/38, middle temporal gyrus, superior temporal gyrus, inferior longitudinal fasciculus | L | -40 8 -26 | 1161 |

Notes: CM=Talairach coordinates of each cluster’s center of mass; L=left; R=right.

**Table S3.** Significant regions of activation for the Group×Phase×Congruency interaction during the Attend-Auditory condition of the multimodal attention task.

| **Region** | **Laterality** | **CM**  **(x,y,z)** | **Volume (µL)** |
| --- | --- | --- | --- |
| Lobule V/VI cerebellum, culmen | R | 15 -48 -14 | 1674 |
| Brodmann Areas 6/4,  precentral gyrus, postcentral gyrus | R | 55 -11 37 | 972 |

Notes: CM=Talairach coordinates of each cluster’s center of mass; L=left; R=right.

**Table S4.** Significant regions of activation for the Group×Visit×Congruency interaction during the Attend-Auditory condition of the multimodal attention task.

| **Region** | **Laterality** | **CM**  **(x,y,z)** | **Volume (µL)** |
| --- | --- | --- | --- |
| Brodmann Areas 13/47,  insula, inferior frontal gyrus | R | 36 22 5 | 1323 |
| Brodmann Areas 10/46,  middle frontal gyrus | L | -29 43 25 | 702 |

Notes: CM=Talairach coordinates of each cluster’s center of mass; L=left; R=right.

**References**

Beers, S. R., Wisniewski, S. R., Garcia-Filion, P., Tian, Y., Hahner, T., Berger, R. P., . . . Adelson, P. D. (2012). Validity of a pediatric version of the Glasgow Outcome Scale-Extended [10.1089/neu.2011.2272 doi]. *J.Neurotrauma*, *29*(6), 1126-1139. PM:22220819

Delis, D. C., Kaplan, E., & Kramer, J. H. (2001). *Delis-Kaplan executive function system (D-KEFS)*. Psychological Corporation.

Denning, J. H. (2012). The efficiency and accuracy of the Test of Memory Malingering trial 1, errors on the first 10 items of the test of memory malingering, and five embedded measures in predicting invalid test performance [acs044 pii ;10.1093/arclin/acs044 doi]. *Arch.Clin.Neuropsychol.*, *27*(4), 417-432. PM:22543569

Derogatis, L. R., & Fitzpatrick, M. (2004). The SCL-90-R, the Brief Symptom Inventory (BSI), and the BSI-18.

Farrar, J. T., Young, J. P., Jr., LaMoreaux, L., Werth, J. L., & Poole, R. M. (2001). Clinical importance of changes in chronic pain intensity measured on an 11-point numerical pain rating scale [S0304-3959(01)00349-9 pii]. *Pain*, *94*(2), 149-158. PM:11690728

Forrest, C. B., Meltzer, L. J., Marcus, C. L., de la Motte, A., Kratchman, A., Buysse, D. J., . . . Bevans, K. B. (2018). Development and validation of the PROMIS Pediatric Sleep Disturbance and Sleep-Related Impairment item banks. *Sleep*, *41*(6). <https://doi.org/ARTN> zsy05410.1093/sleep/zsy054

Genizi, J., Mansour, R., Burbara, M., Gal, S., Nathan, K., Kaly, L., & Yaniv, L. (2025). The HIT-6 Questionnaire Corresponds to the PedMIDAS for Assessment of Pediatric Headaches. *Healthcare*, *13*(23). <https://doi.org/ARTN> 315810.3390/healthcare13233158

Gioia, G. A., Collins, M., & Isquith, P. K. (2008). Improving identification and diagnosis of mild traumatic brain injury with evidence: psychometric support for the acute concussion evaluation [10.1097/01.HTR.0000327255.38881.ca doi ;00001199-200807000-00005 pii]. *J.Head Trauma Rehabil.*, *23*(4), 230-242. <https://doi.org/10.1097/01.HTR.0000327255.38881.ca>

Gioia, G. A., Schneider, J. C., Vaughan, C. G., & Isquith, P. K. (2009). Which symptom assessments and approaches are uniquely appropriate for paediatric concussion? [43/Suppl_1/i13 pii ;10.1136/bjsm.2009.058255 doi]. *Br.J.Sports Med.*, *43 Suppl 1*, i13-i22. <https://doi.org/10.1136/bjsm.2009.058255>

Goodman, R. (1997). The Strengths and Difficulties Questionnaire: a research note. *J.Child Psychol.Psychiatry*, *38*(5), 581-586. PM:9255702

Group, W. (2002). The alcohol, smoking and substance involvement screening test (ASSIST): development, reliability and feasibility. *Addiction*, *97*(9), 1183-1194.

Hergert, D. C., Sicard, V., Stephenson, D. D., Pabbathi, R. S., Robertson-Benta, C. R., Dodd, A. B., . . . Mayer, A. R. (2022). Test-Retest Reliability of a Semi-Structured Interview to Aid in Pediatric Traumatic Brain Injury Diagnosis. *J Int.Neuropsychol.Soc.*, *28*(7), 687-699. <https://doi.org/10.1017/S1355617721000928>

Irwin, D. E., Stucky, B., Langer, M. M., Thissen, D., DeWitt, E. M., Lai, J. S., . . . DeWalt, D. A. (2010). An item response analysis of the pediatric PROMIS anxiety and depressive symptoms scales. *Quality of Life Research*, *19*(4), 595-607. <https://doi.org/10.1007/s11136-010-9619-3>

Kriz, P. K., Stein, C., Kent, J., Ruggieri, D., Dolan, E., O'Brien, M., & Meehan, W. P., III. (2016). Physical Maturity and Concussion Symptom Duration among Adolescent Ice Hockey Players [S0022-3476(15)01516-4 pii ;10.1016/j.jpeds.2015.12.006 doi]. *J.Pediatr.*, *171*, 234-239. PM:26781190

Prinz, R. J., Foster, S., Kent, R. N., & O'Leary, K. D. (1979). Multivariate assessment of conflict in distressed and nondistressed mother-adolescent dyads [10.1901/jaba.1979.12-691 doi]. *J.Appl.Behav.Anal.*, *12*(4), 691-700. PM:541311

Varni, J. W., Seid, M., & Rode, C. A. (1999). The PedsQL: measurement model for the pediatric quality of life inventory. *Med.Care*, *37*(2), 126-139. PM:10024117

Wechsler, D. (2008). *Wechsler adult intelligence scale-fourth*. San Antonio: Pearson.

Wechsler, D. (2014). Wechsler intelligence scale for children - Fifth edition (WISC-V): Technical and interpretive manual. In: Bloomington, MN: Pearson Clinical Assessment.

Wilkinson, G. S., & Robertson, G. J. (2006). *WRAT 4: Wide range achievement test; professional manual*. Psychological Assessment Resources, Incorporated.
